# Domestication-Driven Expansion and Structural Convergence of the Porcine Antiviral Interferon Repertoire

**DOI:** 10.3390/biom16081195

**Published:** 2026-08-17

**Authors:** Jiuyi Li, Niya Tu, Laura C. Miller, Yongming Sang

**Affiliations:** 1 Department of Food and Animal Sciences, College of Agriculture, Tennessee State University, 3500 John A. Merritt Blvd, Nashville, TN 37209, USA; jli4@tnstate.edu (J.L.); tuniyat@outlook.com (N.T.); 2 Diagnostic Medicine and Pathobiology, College of Veterinary Medicine, Kansas State University, 1800 Denison Ave, Manhattan, KS 66506, USA; lauramiller@ksu.edu

**Keywords:** porcine interferons, comparative genomics, domestication, AlphaFold2, structural evolution, purifying selection, livestock immunogenetics

## Abstract

The porcine interferon (IFN) system is highly diversified, particularly within Type I subfamilies, yet its evolutionary trajectory across domestication and breed formation remains poorly characterized. We performed a comprehensive comparative genomic and structural analysis of 432 IFN sequences spanning all IFN types across 11 *Sus scrofa* breeds representing commercial, indigenous, and wild/outgroup lineages. Phylogenetic reconstruction, pairwise dN/dS selection pressure analysis, and AlphaFold2-based 3D structure prediction coupled with DALI structural similarity mapping were integrated to resolve repertoire architecture, evolutionary constraints, and domestication-associated divergence. IFN repertoire organization is governed primarily by family identity rather than breed origin, with Type I IFN-α, -δ, and -ω subfamilies showing pronounced gene expansion in domestic breeds. Phylogenetic clustering and structural similarity consistently grouped sequences by subtype, independent of domestication history. Pervasive purifying selection (median ω = 0.48) maintained functional constraints across all lineages. Commercial breeds exhibited significantly higher within-category structural convergence alongside expanded repertoires, while structural conservation was evolutionarily decoupled from sequence-level selective pressure. Domestication potentially drove coordinated IFN repertoire expansion and structural conservation with related purifying selection at the gene level. These findings establish a genomic framework linking breed-specific IFN architecture to antiviral capacity and provide a foundation for immunogenetic-informed breeding strategies in the swine model.

## 1. Introduction

Interferons (IFNs) are a family of secreted cytokines that constitute a central pillar of innate immune defense in higher vertebrates. They are rapidly produced in response to viral infections and other pathogen-associated stimuli and were first discovered through their antiviral interference against influenza infection in 1957. Since their discovery, extensive studies have demonstrated that IFNs play critical roles in antiviral defense, antitumor activity, and immunomodulatory regulation. IFNs are classified into three major types based on receptor usage and structural features: Type I IFNs (comprising IFN-α, IFN-β, IFN-ω, IFN-αω, IFN-δ, IFN-ε, and IFN-κ among others) signal through the heterodimeric IFNAR1/IFNAR2 receptor complex; Type II IFN (IFN-γ) acts through IFNGR1/IFNGR2; and the Type III IFN-λ family signals via the IFNLR1/IL10RB receptor. Despite their shared role in host defense, these IFN families diverge substantially in expression patterns, tissue distribution, receptor specificity, and biological function, making the IFN gene system a compelling model for studying the evolution of immune defense across vertebrate lineages [1,2,3].

Compared with other mammals, swine (*Sus scrofa*) display a notably expanded and diversified Type I IFN repertoire, particularly within the multi-gene subfamilies including IFN-α, IFN-δ, and IFN-ω subtypes. This diversity likely reflects the strong selective pressure imposed by the wide range of infectious pathogens, including influenza virus, African swine fever virus, porcine reproductive and respiratory syndrome virus (PRRSV), and others, to which swine are exposed throughout their evolutionary and especially domestic history [4,5,6]. As a globally critical livestock species with profound relevance to animal health, food security, and zoonotic disease surveillance, understanding the functional architecture of the porcine IFN system carries both basic and applied significance. Moreover, the biological diversity of the swine IFN repertoire makes this species a particularly powerful comparative model for investigating how immune gene families expand, contract, and functionally diverge under natural and artificial selection [7,8,9,10].

Modern swine populations encompass a broad spectrum of genetic and ecological backgrounds, including commercial breeds subjected to intensive artificial selection [11], such as Duroc, Large white, Landrace, Berkshire, indigenous breeds shaped by geographic isolation and local adaptation, and lineages with closer affinity to related wild/outgroup ancestors. These populations have experienced distinct combinations of domestication history, breeding regimes, and pathogen exposure, each of which may leave signatures in immune gene architecture through mechanisms including gene duplication or loss, sequence divergence, and shifts in functional specialization [4,12,13]. Consequently, swine breeds may differ not only in production traits and disease susceptibility phenotypes, but also in the composition, copy number, and sequence diversity of their IFN gene repertoires. Despite this potential diversity, systematic comparative studies across swine breeds are rare [14,15,16].

Here, we address this gap by performing a comprehensive comparative genomic analysis of IFN gene repertoires across 11 swine breeds spanning commercial domestic breeds, indigenous breeds, and feral/wild-associated lineages. We characterized breed-level variation in IFN gene copy number, paralog diversity, and sequence evolution, with particular focus on the expanded Type I IFN subfamilies [17]. Phylogenetic reconstruction was used to resolve evolutionary relationships among IFN paralogs across breeds, and selection pressure analysis was applied to assess whether individual IFN genes have evolved under positive, neutral, or purifying selection in different lineages. To extend beyond sequence-level inference, we further employed AlphaFold2 to predict the three-dimensional structures of IFN proteins across breeds and applied DALI-based structural similarity analysis to evaluate the degree of structural conservation or divergence among paralogs and orthologs [18,19]. Together, these genomic, phylogenetic, and structural analyses allow us to ask both how domestication and artificial selection have shaped IFN repertoire architecture, and conversely, whether the diversity of IFN gene repertoires correlates with inter-breed differences in antiviral immune capacity [20,21]. Our findings provide a multi-dimensional view of immunogenetic diversification in swine and establish a framework for linking IFN genomic variation to breed-level differences in IFN-mediated immune defense.

## 2. Materials and Methods

### 2.1. Genome Assembly Selection and Breed Coverage

A total of 11 *Sus scrofa* genome assemblies representing breeds spanning three continents were selected for this study, encompassing commercial Western breeds, Asian indigenous breeds, and wild boar (Bushpig & Visayan warty pig) as evolutionary outgroups (Table 1). Duroc, Large White, and Landrace are breeds of Western genetic origin; however, they were classified as commercial breeds because they are now globally distributed and widely used as standardized genetic foundations of intensive pig production, including in Asian commercial systems. The commercial–indigenous comparison therefore represents differences in production and breeding history, although it also partially overlaps with Western–Asian phylogeographic background. PB115 and *Sus cebifrons* were included as a wild/outgroup reference category. PB115 represents a wild-related *Sus scrofa* lineage, whereas *Sus cebifrons* is a distinct species within the genus *Sus*. Their inclusion in the same reference category was intended to provide a broader non-domestic evolutionary comparison and should not be interpreted as indicating that the two lineages are taxonomically equivalent or that *S. cebifrons* is a wild ancestral form of domestic *S. scrofa*. All domestic breed assemblies were aligned and annotated with reference to the *Sus scrofa* reference genome assembly v11.1 (*Sscrofa11.1*; GCA_000003025.6) [22]. This reference was chosen for its high chromosomal contiguity and comprehensive gene annotation, providing a stable coordinate framework for cross-breed comparisons.

### 2.2. Interferon Gene Identification and Sequence Extraction

IFN gene and protein sequences were systematically identified across all 11 breed assemblies, targeting all nine recognized IFN subfamilies in swine: IFN-α, IFN-αω, IFN-β, IFN-γ, IFN-δ, IFN-ε, IFN-κ, IFN-λ, and IFN-ω. Gene identification was performed using a combination of NCBI BLAST-based homology searches against known porcine IFN sequences and synteny-guided annotation relative to the *Sscrofa11.1* reference. Candidate loci were inspected manually to confirm gene structure, reading frame integrity, and the absence of premature stop codons. Pseudogenes and truncated sequences were flagged and excluded from downstream protein-level analyses. In total, 432 IFN gene/protein sequences were extracted across all breeds and families, constituting the primary dataset for this study.

### 2.3. Quality Control and Sequence Filtering

To ensure the integrity of structural and evolutionary analyses, all extracted sequences were subjected to a minimum length threshold of 130 amino acid residues. This cutoff was established to exclude highly truncated or partially assembled sequences that would otherwise introduce artifacts in structural prediction and pairwise evolutionary rate estimation. Sequences failing this criterion were recorded but excluded from phylogenetic, dN/dS, and structural analyses. The final curated dataset was verified for completeness of the signal peptide and mature protein regions where applicable.

### 2.4. Multiple Sequence Alignment and Phylogenetic Analysis

Protein sequences spanning all IFN families were aligned using MAFFT v7 [23]. Phylogenetic trees were constructed using IQ-TREE v3.1.2 under the best-fit substitution model automatically selected by ModelFinder Plus based on the Bayesian Information Criterion [24,25]. The best-fit model selected was Q.BIRD+G4. Branch support was evaluated using 1000 replicates of the ultrafast bootstrap approximation (UFBoot2) combined with the SH-like approximate likelihood ratio test (SH-aLRT), with bootstrap trees additionally refined by nearest-neighbor interchange (NNI) searches. Branches with SH-aLRT ≥ 80% and UFBoot ≥ 95% were considered well-supported [26]. Phylogenetic visualization and annotation were performed using the Interactive Tree of Life iTOL v7 platform, with IFN family designations and breed-of-origin labels retained to facilitate identification of lineage-specific clustering or diversification patterns [27].

### 2.5. Selection Pressure Analysis (dN/dS)

To assess the mode of molecular evolution acting on porcine IFN genes, pairwise synonymous (dS) and nonsynonymous (dN) substitution rates were estimated for all sequence pairs using a custom Python v.3.8.8 pipeline implementing the Nei–Gojobori method (NG86) with Jukes–Cantor correction. Prior to calculation, codon alignments were generated using a protein-guided approach based on the BLOSUM62 substitution matrix. The dN/dS ratio (ω) was used as an index of selective pressure: values of ω < 1 indicate purifying (negative) selection, ω ≈ 1 indicates neutral evolution, and ω > 1 is consistent with positive (diversifying) selection.

To characterize selection across multiple biological levels, pairwise ω values were examined from several complementary perspectives. All-versus-all pairwise dN/dS values were computed across the full sequence dataset and summarized as heatmaps with sequences ordered by breed, as well as dN versus dS scatter plots and ω frequency distribution histograms. Within-breed paralog heatmaps were constructed for each breed to identify gene pairs exhibiting the greatest synonymous divergence among paralogs. Between-breed ortholog heatmaps were generated as breed-by-breed matrices of median ω values across shared orthologs, revealing which breed pairs are most or least divergent. Per-gene and per-family summaries were further derived as barplots and boxplots, respectively, to compare mean ω across IFN subtypes and to contrast selection pressures among Type I, Type II, and Type III IFN families. Where structural data were available, pairwise ω values were plotted against DALI Z-scores to evaluate whether structural conservation is reflected in the degree of sequence-level purifying selection.

To validate the robustness of the NG86-based pairwise dN/dS estimates, a subset of orthologous same-gene comparisons was reanalyzed using the Yang–Nielsen method implemented in the yn00 program of PAML v4.10.10. Codon alignments for these comparisons were supplied to yn00, and resulting dN, dS, and ω estimates were compared with the corresponding NG86 estimates by linear regression. Concordance between the two methods was used as an independent validation of the pairwise selection-pressure estimates reported in the main analysis [28].

### 2.6. Three-Dimensional Structure Prediction with AlphaFold2

To evaluate structural conservation and divergence beyond primary sequence similarity, three-dimensional structures of all curated IFN protein sequences were predicted using AlphaFold2 [13]. Each IFN protein sequence was modeled individually using the AlphaFold2_batch pipeline with automatically generated multiple sequence alignments (MSAs). Model confidence was evaluated using predicted local distance difference test (pLDDT) scores. Predicted structures with mean pLDDT values ≥ 70 across the mature protein region were retained for downstream structural similarity analyses. In addition, predicted structures shorter than 130 amino acids were classified as low-completeness structural models but were retained in the downstream structural analyses and interpreted cautiously as potential structural outliers.

### 2.7. Structural Similarity Analysis with DALI

Pairwise structural similarity among predicted IFN protein structures was evaluated using a local implementation of DALI v5 [29], which compares intramolecular residue–residue distance matrices to identify conserved structural cores and generate optimal three-dimensional superimpositions while accommodating local insertions, deletions, and geometric distortions. DALI Z-scores and Cα RMSD values were computed for all within- and between-family pairwise comparisons; Z-scores ≥ 2 were considered indicative of statistically significant structural similarity. Resulting structural distance matrices were used to construct similarity dendrograms, which were then compared with sequence-based phylogenies to identify instances of structural conservation despite substantial sequence divergence, as well as structural diversification among closely related paralogs. Because protein tertiary structure is generally more evolutionarily conserved than primary sequence, this structural framework provides a complementary and biologically meaningful perspective on IFN evolutionary relationships, particularly with respect to receptor-binding interfaces and signaling-associated structural motifs.

### 2.8. Comparative Analysis Across Breed Categories

To investigate the relationship between domestication history and IFN repertoire architecture, breeds were classified into three categories based on breeding history: (1) commercial breeds subjected to intensive artificial selection (Duroc, Landrace, Large White; *n* = 3), (2) indigenous breeds with extended geographic isolation and limited formal selection (Meishan, Juema, Wuzhishan, Nero Siciliano, Nanchukmacdon, Ninghe; *n* = 6), and (3) wild or ancestral-proxy breeds representing the pre-domestication state (*Sus cebifrons* and PB115; *n* = 2). Three metrics were compared across categories: IFN gene copy number per breed, within-category pairwise dN/dS from ortholog comparisons, and within-category DALI structural similarity Z-scores. Given the non-normal distribution of pairwise values, statistical comparisons were performed using the Kruskal–Wallis H test, followed by post hoc Mann–Whitney U tests for pairwise group comparisons. Significance thresholds were set at *p* < 0.05 (*), *p* < 0.01 (**), and *p* < 0.001 (***).

To examine whether sequence-level selective pressure and structural conservation are coupled across IFN families, pairwise DALI Z-scores were matched to corresponding dN/dS values using a name-normalization pipeline that reconciled naming differences between the DALI structural matrix (432 structures) and the dN/dS dataset (432 sequences), achieving an 89.6% match rate (83,028 matchable pairs). Matched pairs were classified as between-family comparisons (*n* = 57,109), within-family paralogs (*n* = 18,816), and cross-breed orthologs (*n* = 1056). Linear regression was applied to assess the strength of association, with R^2^ and slope reported for both the full dataset and the ortholog subset independently.

## 3. Results

### 3.1. IFN Gene Repertoire Composition Across Swine Breeds

**Figure 1:**
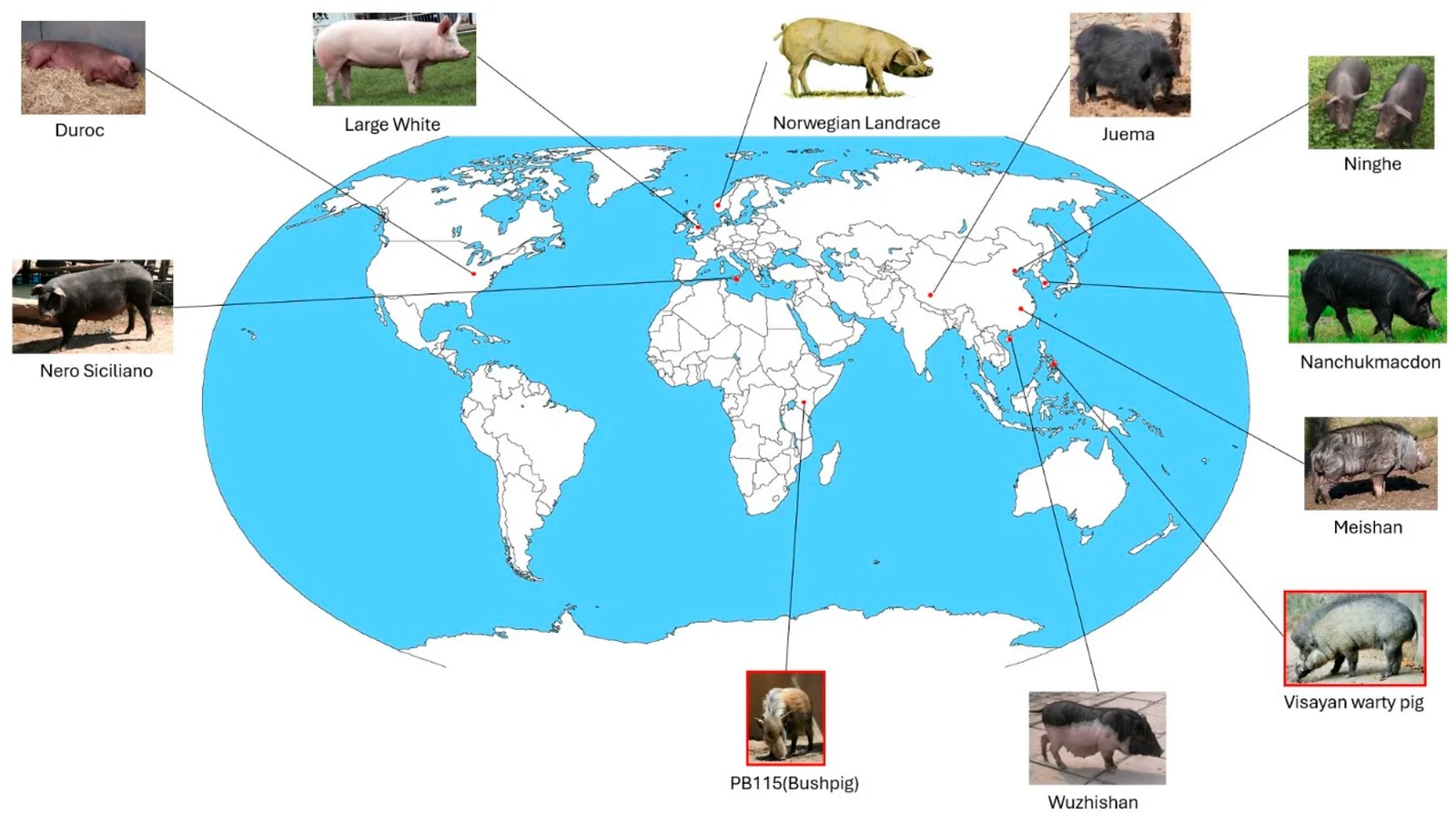
Geographic origins of the 11 *Sus scrofa* breeds and related suids used in this study. World map showing the sampling locations of pig breeds included in the comparative genomic analysis. Commercial breeds are represented by Duroc (United States), Large White (United Kingdom), and Norwegian Landrace (Norway). European heritage breeds include Nero Siciliano (Italy/Sicily). Asian indigenous breeds are represented by Juema, Ninghe, Nanchukmacdon, Meishan, Wuzhishan. PB115 (Bushpig; *Potamochoerus larvatus*) from sub-Saharan Africa and Visayan warty pig (*Sus cebifrons*) from the Philippines (both highlighted with red borders) were included as outgroup taxa. Lines connect each breed photograph to its approximate geographic origin on the map. Red dots on the map indicate sampling locations.

**Figure 2:**
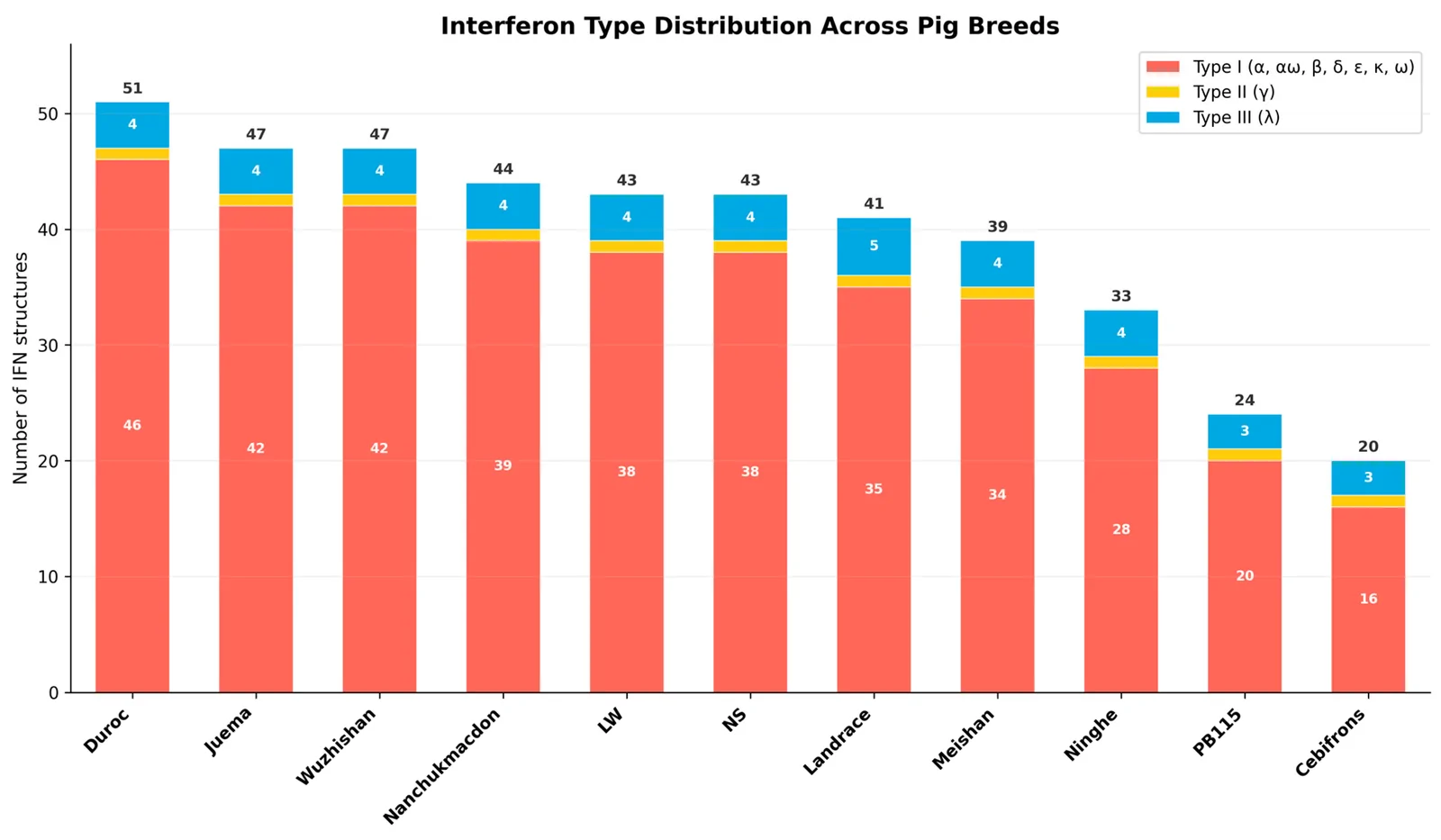
Distribution of functional IFN gene numbers across 11 pig breeds and one outgroup taxon. Stacked bar chart showing the total number of functional IFN genes identified in each breed, categorized by IFN type. Type I IFNs (α, β, δ, ε, κ, ω, αω; red) constitute the largest proportion in all breeds, followed by Type III IFNs (λ; blue) and Type II IFN (γ; yellow). Breeds are arranged in descending order of total IFN gene count. Duroc had the highest total (*n* = 51), while *Sus cebifrons* had the lowest (*n* = 20). PB115 (Bushpig) and *Cebifrons* were included as outgroup taxa. Numerical labels within each bar segment or above the bar indicate the gene count of each IFN type or total IFN, respectively. LW, Large White; NS, Nero Siciliano.

To characterize the interferon gene repertoire across diverse swine lineages, we systematically identified and curated IFN gene sequences from 11 *Sus scrofa* genome assemblies representing breeds distributed across three continents (Figure 1). The breed panel encompassed three commercial Western breeds subjected to intensive artificial selection (Duroc, Large White, Norwegian Landrace), six indigenous or regionally adapted breeds (Juema, Ninghe, Nanchukmacdon, Nero Siciliano, Meishan, Wuzhishan), and two wild or ancestral-proxy lineages serving as pre-domestication outgroups (PB115/Bushpig and Visayan warty pig, *Sus cebifrons*). Following quality filtering with a minimum length threshold of 130 amino acid residues and exclusion of pseudogenes, a total of 432 IFN protein sequences were retained spanning all 9 recognized IFN families and 56 distinct subtypes (Figure 2).

Total IFN gene counts varied across breeds, ranging from 51 sequences in Duroc to 20 in *Sus cebifrons*. Among domestic breeds, Duroc harbored the largest repertoire, followed by Juema and Wuzhishan (both *n* = 47), Nanchukmacdon (*n* = 44), Large White and Nero Siciliano (both *n* = 43), Landrace (*n* = 41), Meishan (*n* = 39), and Ninghe (*n* = 33). The two wild-proxy lineages consistently showed the smallest total repertoires (PB115, *n* = 24; and *Sus cebifrons*, *n* = 20).

Type I IFNs constituted the dominant component of the repertoire in every breed examined (Figure 2). Within Type I IFNs, three subfamilies showed pronounced multi-gene representation: IFN-α (*n* = 147 across all breeds), IFN-δ (*n* = 116), and IFN-ω (*n* = 71), collectively accounting for 77.3% of all sequences in the dataset. Five subfamilies were represented by exactly one gene per breed in all assemblies: IFN-β (*n* = 11), IFN-γ (*n* = 11), IFN-ε (*n* = 11), IFN-αω (*n* = 11), and IFN-κ (*n* = 11). IFN-λ showed intermediate gene number variation across breeds (total *n* = 43; range 3–5 per breed). Type II (IFN-γ) conserves a single gene across all 11 breeds, while inter-breed variation in total IFN count is primarily attributed to differences within multi-gene subtypes of IFN-α, IFN-δ, and IFN-ω.

### 3.2. Phylogenetic Relationships Among Porcine IFN Proteins

**Figure 3:**
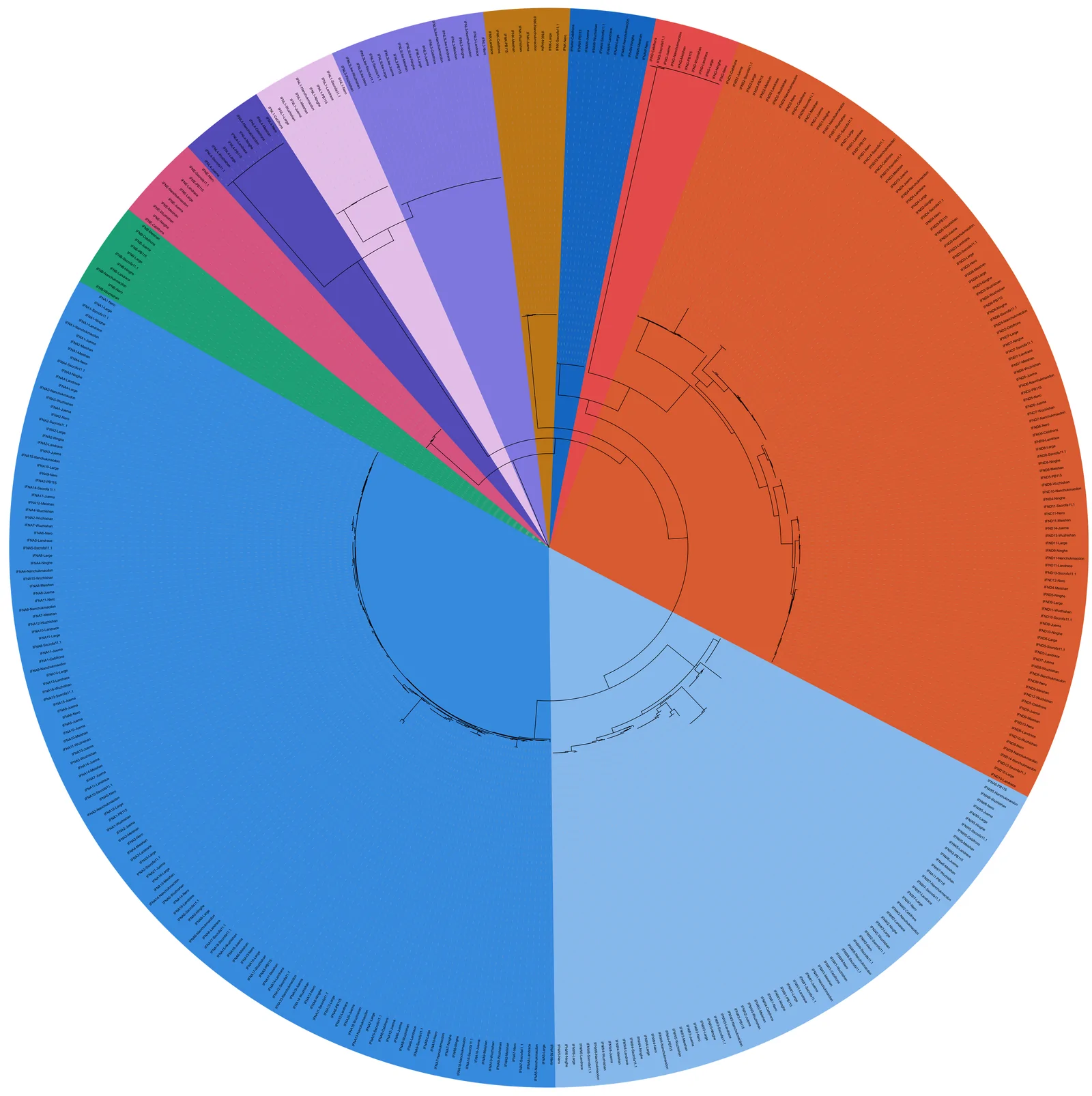
Maximum-likelihood phylogenetic tree of porcine interferon (IFN) family members across 11 breeds and two outgroup taxa based on amino acid sequences. The phylogenetic tree was constructed using IQ-TREE based on multiple sequence alignment of deduced amino acid sequences from all IFN gene members identified across nine *Sus scrofa* breeds: Duroc (*Sscrofa11.1*), Juema, Wuzhishan, Nanchukmacdon, Large White [LW], Nero Siciliano [NS], Norwegian Landrace, Meishan, and Ninghe, and two outgroup taxa (PB115/Bushpig and *Sus cebifrons*. Each tip label indicates the IFN gene name followed by the breed of origin (e.g., IFNA1-Duroc). Sequences are grouped into major IFN types: Type I IFNs including IFN-α (IFNA), IFN-β (IFNB), IFN-δ (IFND), IFN-ε (IFNE), IFN-κ (IFNK), IFN-ω (IFNW), and IFN-αω (IFNAW); Type II IFN-γ (IFNG); and Type III members IFN-λ1 (IFNL1), IFN-λ3 (IFNL3), IFN-λ3-like (IFNL3Like), and IFN-λ4 (IFNL4). Colored background sectors distinguish the different IFN gene subfamilies and their corresponding phylogenetic clades. Orthologous sequences from different breeds cluster together within each gene clade, reflecting conservation of IFN gene identity across breeds. Scale bar represents one amino acid substitution per site.

Maximum-likelihood phylogenetic analysis of 432 curated porcine IFN protein sequences under the Q.BIRD+G4 substitution model recovered a topology in which sequences partitioned into nine major clades corresponding to the nine IFN subfamilies (Figure 3). Family-level separation was supported by long inter-clade branches, consistent with the deep evolutionary divergence between IFN types. Within each family clade, the primary axis of clustering was subtype identity rather than breed of origin: across all families, sequences from different breeds were consistently interleaved within subtype-level sub-clades rather than segregating by breed, indicating that orthologous subtype relationships predate breed divergence. Due to the high sequence similarity among recently duplicated paralogs within the multi-gene IFN subtypes, many within-family nodes showed short branch lengths and moderate bootstrap support, a pattern consistent with rapid and recent paralog expansion rather than phylogenetic ambiguity in the deeper family-level topology.

The IFN-α clade was the largest and most internally structured, comprising 21 numbered subtypes (IFNA1–IFNA21) with between 1 and 11 sequences per subtype. Subtypes IFNA19, IFNA20, and IFNA21 were each represented by a single sequence from one breed, suggesting either breed-specific gene duplications or incomplete detection in other assemblies. The IFN-ω clade resolved into nine subtypes (IFNW1–IFNW9), with IFNW9 likewise represented by a single sequence, and the IFN-δ clade into fifteen subtypes (IFND1–IFND15), with IFND13 and IFND15 each represented by two or fewer sequences. In all three multi-gene IFN subtypes, the two wild-proxy lineages (PB115, *Sus cebifrons*) were present in only a subset of subtype sub-clades, consistent with their reduced total repertoire sizes.

The five single-gene Type I families, IFN-β, IFN-ε, IFN-κ, IFN-γ, and IFN-αω, each formed compact clades of exactly 11 sequences with minimal internal branch lengths, reflecting high sequence conservation across all 11 breeds. IFN-γ similarly resolved as a tight clade of 11 sequences. The Type III IFN-λ clade comprised three subtypes (IFNL1, IFNL3, IFNL4), with IFNL3 and IFNL3-like forming sister sub-clades consistent with a recent lineage-specific duplication. At the deepest level, all seven Type I IFN families formed a superordinate clade clearly separated from both IFN-γ and IFN-λ, consistent with the independent evolutionary origins of the three IFN types.

### 3.3. Porcine IFN Genes Evolve Predominantly Under Purifying Selection

**Figure 4:**
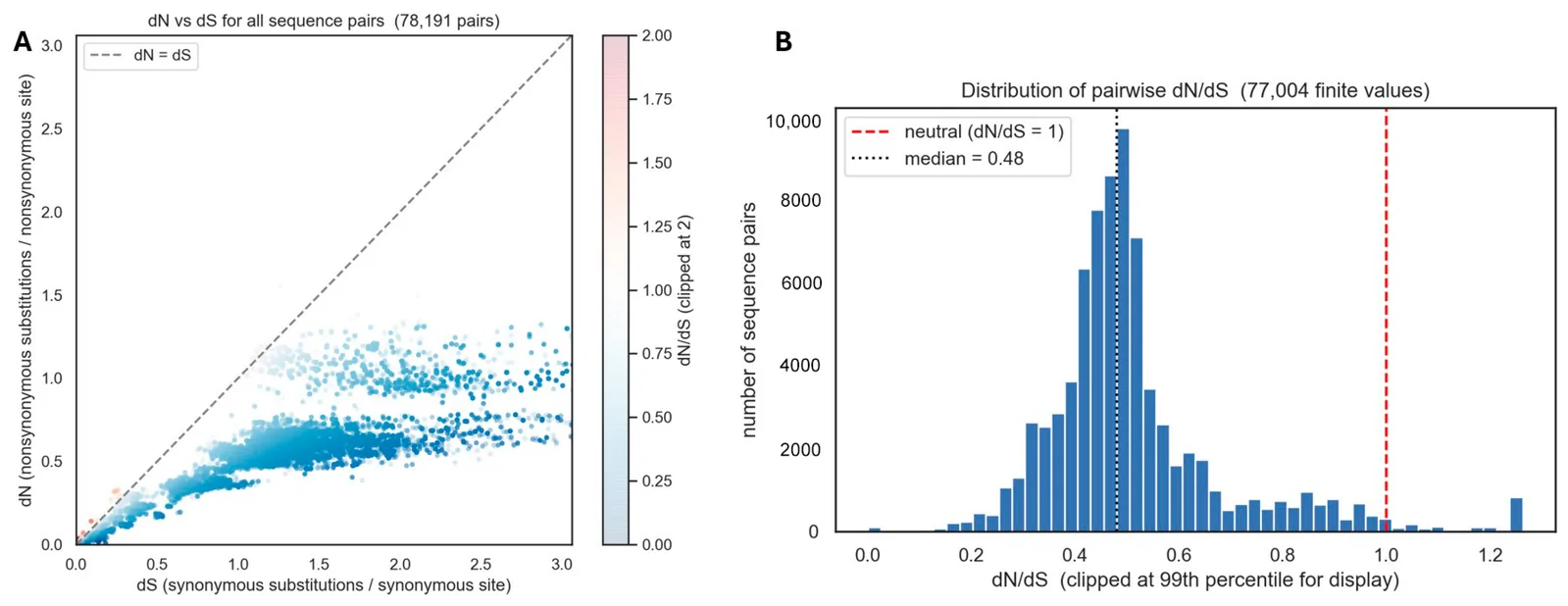
Pairwise synonymous and nonsynonymous substitution rates across all IFN gene sequence comparisons. (**A**) Scatter plot of dN (nonsynonymous substitutions per nonsynonymous site) versus dS (synonymous substitutions per synonymous site) for all 78,191 pairwise sequence comparisons. Each point is color-coded by the dN/dS ratio (ω), clipped at 2.0 for display clarity. The dashed diagonal line represents the neutral expectation (dN = dS; ω = 1). The majority of points fall below this line, indicating predominant purifying selection acting on porcine IFN genes. (**B**) Frequency distribution of pairwise dN/dS values from 77,004 finite-value comparisons, with values clipped at the 99th percentile for visualization. The dotted black vertical line indicates the overall median dN/dS of 0.48, and the dashed red vertical line marks the neutral threshold (dN/dS = 1). The right-skewed distribution centered well below 1.0 is consistent with pervasive purifying selection across the porcine IFN gene family. dN/dS values were computed using the Nei–Gojobori (NG86) method.

To assess the mode of molecular evolution acting on porcine IFN genes, pairwise dN/dS ratios (ω) were computed for all 78,191 sequence pairs across the full 432-sequence dataset. Of these, 77,004 pairs yielded finite ω values and were retained for downstream analysis. The dN versus dS scatter plot (Figure 4A) showed that the large majority of sequence pairs fell below the dN = dS diagonal, indicating that nonsynonymous substitutions accumulate more slowly than synonymous substitutions across the IFN repertoire as a whole. The distribution of pairwise ω values (Figure 4B) was unimodal and right-skewed, with a median of 0.48 and a mode centered near 0.45–0.50. The vast majority of pairs fell below ω = 1, with only a small fraction of pairs in the tail of the distribution exceeding the neutral threshold, visible as a minor peak beyond the red dashed line in Figure 4B. To confirm that these patterns were not dependent on the dN/dS estimation method, a subset of same-gene pairwise comparisons was reanalyzed using the Yang–Nielsen method implemented in yn00 from PAML v4.10.10. YN00 estimates showed strong concordance with the Nei–Gojobori estimates used in the main analysis (R^2^ = 0.894, slope = 0.935, *n* = 1056 matched finite pairs; Supplementary Figure S1), supporting the robustness of the inferred genome-wide pattern of purifying selection. Sliding-window dN/dS analysis of representative IFN orthologs further supported the predominance of purifying selection across the porcine IFN repertoire. Representative genes included Type I IFNs IFN-α (IFNA1), IFN-β (IFNB), IFN-δ (IFND3), IFN-κ (IFNK), and IFN-ω (IFNW1), as well as Type II IFN-γ (IFNG). Across these genes, all informative codon windows showed ω values below 1.0, consistent with the pairwise dN/dS results. Several genes showed localized windows with modestly elevated ω values, but these remained below the neutral threshold, suggesting regional variation in selective constraint rather than positive selection (Figure S2).

**Figure 5:**
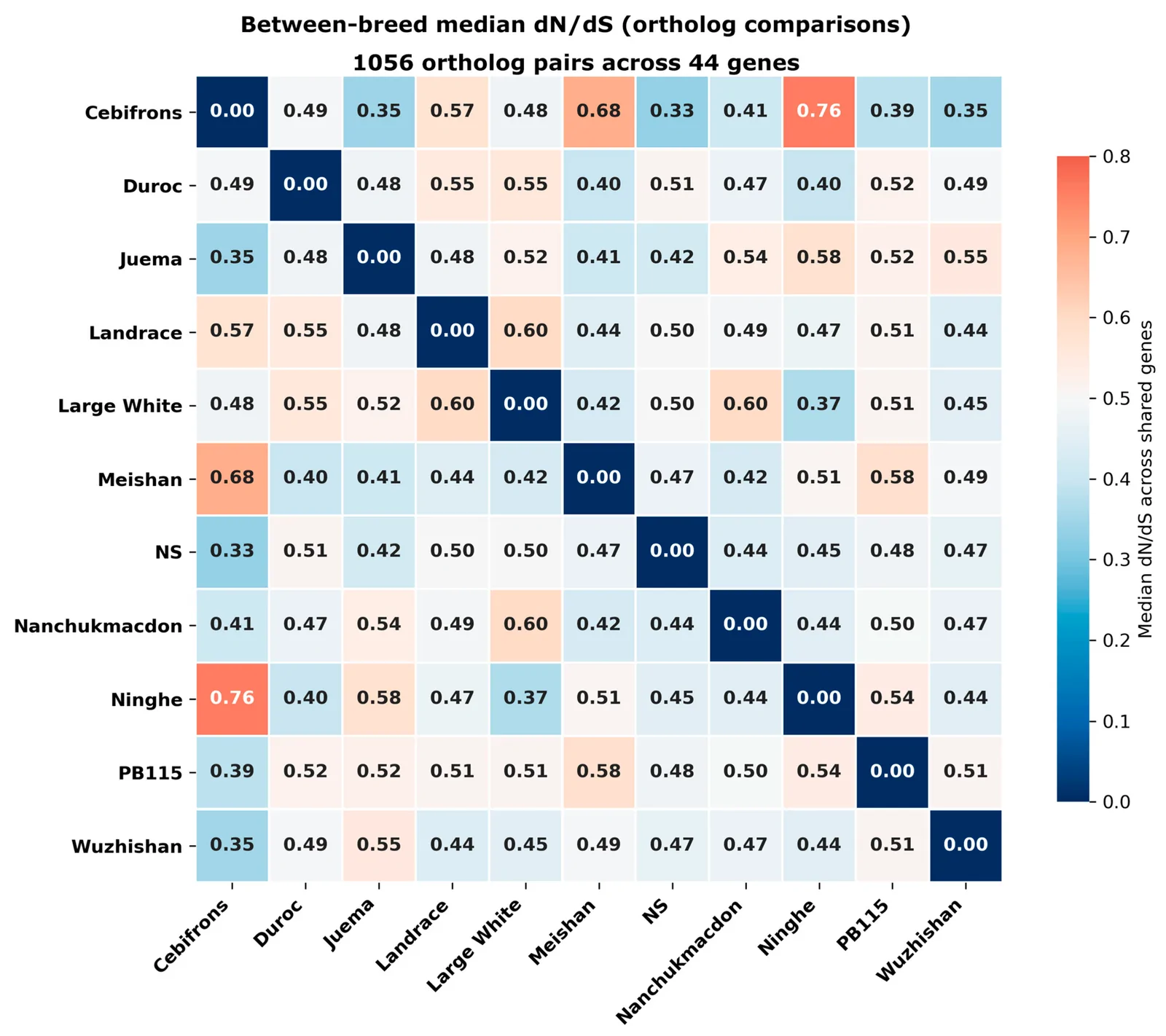
Between-breed median dN/dS values for orthologous IFN gene pair comparisons. Symmetric heatmap displaying the median dN/dS ratio calculated from 1056 orthologous pairwise comparisons across 44 IFN genes for each breed pair. Color scale ranges from dark blue (low dN/dS, strong purifying selection) to red-orange (elevated dN/dS, relaxed constraint or positive selection). Diagonal cells (self-comparisons) are set to 0.00 (dark blue). The highest between-breed median dN/dS values were observed for the Ninghe–*Cebifrons* (0.76) and Meishan–*Cebifrons* (0.68) pairs, reflecting greater divergence between Chinese indigenous breeds and the Sus *Cebifrons* outgroup. Most breed pairs showed median dN/dS values between 0.40 and 0.60, consistent with moderate purifying selection. NS, Nero Siciliano; PB115, Bushpig; LW, Large White.

Between-breed ortholog comparisons across 1056 ortholog pairs spanning 44 shared genes revealed a narrow range of median ω values across breed pairs, spanning from 0.33 to 0.76 (Figure 5). The lowest median ω was observed between Nero Siciliano and *Sus cebifrons* (ω = 0.33), between Juema and *Sus cebifrons* (ω = 0.35), and between Wuzhishan and *Sus cebifrons* (ω = 0.35). The highest median ω values were observed between Ninghe and *Sus cebifrons* (ω = 0.76) and between Meishan and *Sus cebifrons* (ω = 0.68), representing the most divergent breed pairs in the dataset. Among purely domestic breed comparisons, median ω values were generally clustered in the range of 0.40–0.60, with Large White versus Landrace (ω = 0.60), Large White versus Nanchukmacdon (ω = 0.60), and Large White versus Meishan (ω = 0.60) among the higher values observed. Comparisons involving Juema showed consistently lower median ω values against most other breeds (range 0.41–0.55), suggesting relatively constrained ortholog divergence in this breed. No breed pair exceeded a median ω of 1.0, confirming that purifying selection is the predominant evolutionary regime across orthologous IFN gene comparisons at the breed level.

### 3.4. AlphaFold2 Structural Prediction Reveals High-Confidence IFN Fold Models

**Figure 6:**
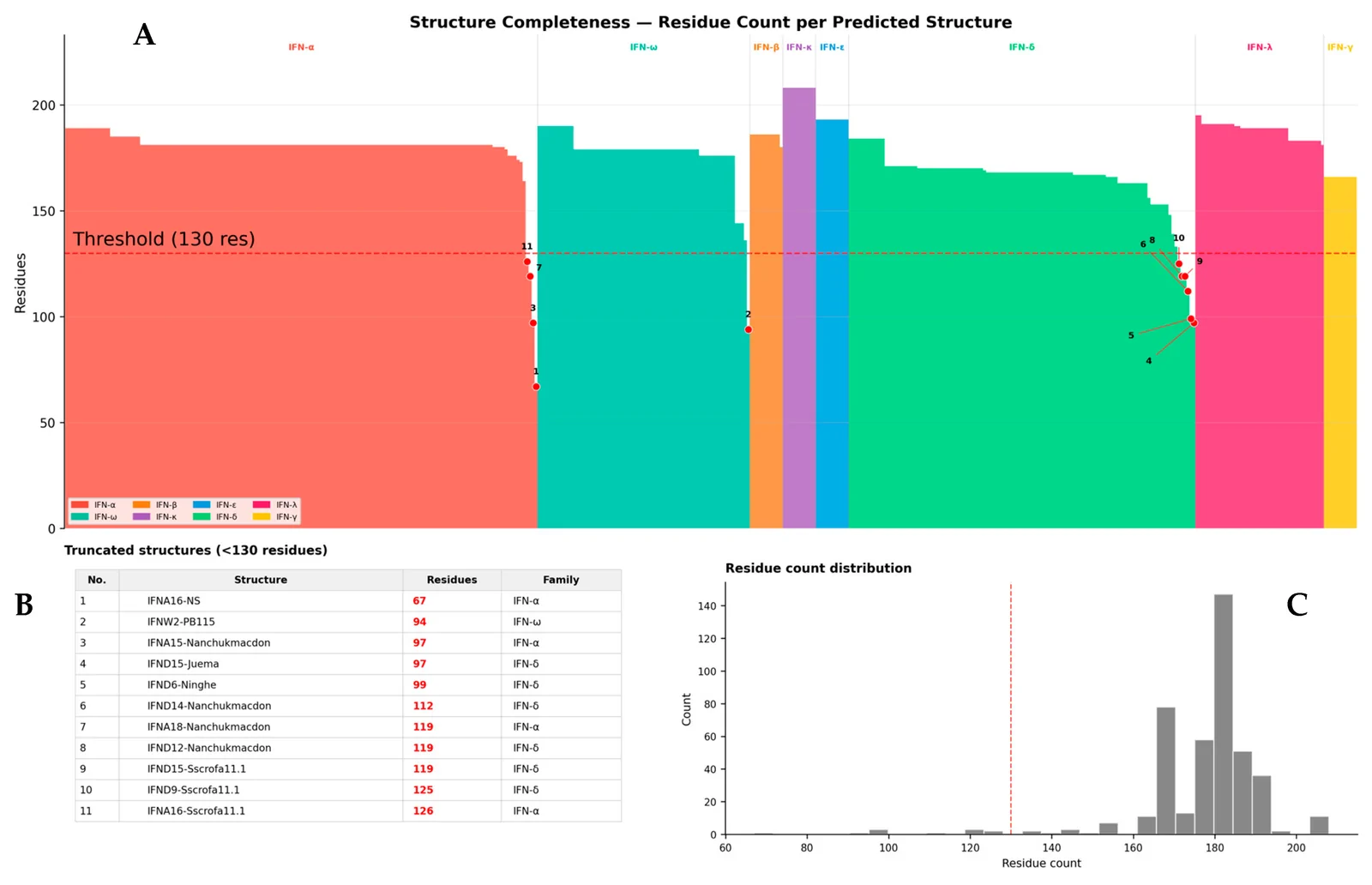
Quality assessment of predicted IFN protein structures based on residue completeness. (**A**) Bar chart showing the residue count for each AlphaFold 2-predicted IFN structure, grouped and color-coded by IFN family: IFN-α (red), IFN-ω (teal), IFN-β (orange), IFN-κ (yellow-green), IFN-ε (purple), IFN-δ (green), IFN-λ (pink), and IFN-γ (gold). Structures are arranged in descending order of residue count within each family. The red dashed horizontal line indicates the completeness threshold of 130 residues; numbered red points identify the 11 structures falling below this cutoff. (**B**) Table listing all 11 truncated structures with residue counts highlighted in red, along with their gene-breed identifiers and IFN family assignments. These 11 structures were flagged as low-completeness structural models but were retained in the all-against-all DALI analysis to permit identification of truncation-associated structural outliers. Their results were interpreted cautiously in subsequent structural comparisons. (**C**) Histogram of residue count distribution across all predicted structures; the red dashed vertical line marks the 130-residue quality threshold. The distribution is right-skewed with the majority of structures concentrated between 160–190 residues, reflecting the characteristic length of mature porcine IFN proteins, while the small left tail corresponds to the truncated sequences identified in panels A and B. NS, Nero Siciliano; PB115, Bushpig.

Three-dimensional protein structures were predicted for all 432 curated IFN sequences using AlphaFold2. Assessment of structural completeness based on predicted residue count (Figure 6A) identified 11 structures falling below the 130-residue quality threshold, listed in Figure 6B with residue counts ranging from 67 (IFNA16-Nero Siciliano) to 126 (IFNA16-*Sscrofa11.1*). These 11 truncated structures spanned two families: seven belonged to IFN-δ (IFND6-Ninghe, IFND9-*Sscrofa11.1*, IFND12-Nanchukmacdon, IFND14-Nanchukmacdon, IFND15-Juema, IFND15-*Sscrofa11.1*, IFND9-*Sscrofa11.1*) and four to IFN-α (IFNA15-Nanchukmacdon, IFNA16-Nero Siciliano, IFNA16-*Sscrofa11.1*, IFNA18-Nanchukmacdon). The remaining 421 structures passed the completeness threshold, whereas the 11 structures below the threshold were retained as flagged low-completeness models for sensitivity and outlier assessment. Accordingly, all 432 structures were included in the DALI similarity matrix. The residue count distribution of the full dataset (Figure 6C) was bimodal, with the large majority of structures concentrated between 160 and 190 residues, a secondary cluster near 130–140 residues corresponding to the IFN-δ family, and the 11 truncated outliers forming a sparse low-residue tail below the threshold.

### 3.5. Structural Conservation Mirrors IFN Family Classification but Is Decoupled from Breed Identity

Pairwise structural similarity among all 432 predicted IFN protein structures was evaluated using DALI, generating a total of 93,096 pairwise Z-score comparisons. Z-scores ranged from 0.1 to 33.9 across the full dataset, with a mean off-diagonal Z-score of 17.2 and a median of 19.6. Of all pairwise comparisons, 68,699 pairs (73.8%) exceeded the significance threshold of Z ≥ 2, while 3014 pairs (3.2%) fell below Z = 2, indicating no detectable structural similarity.

**Figure 7:**
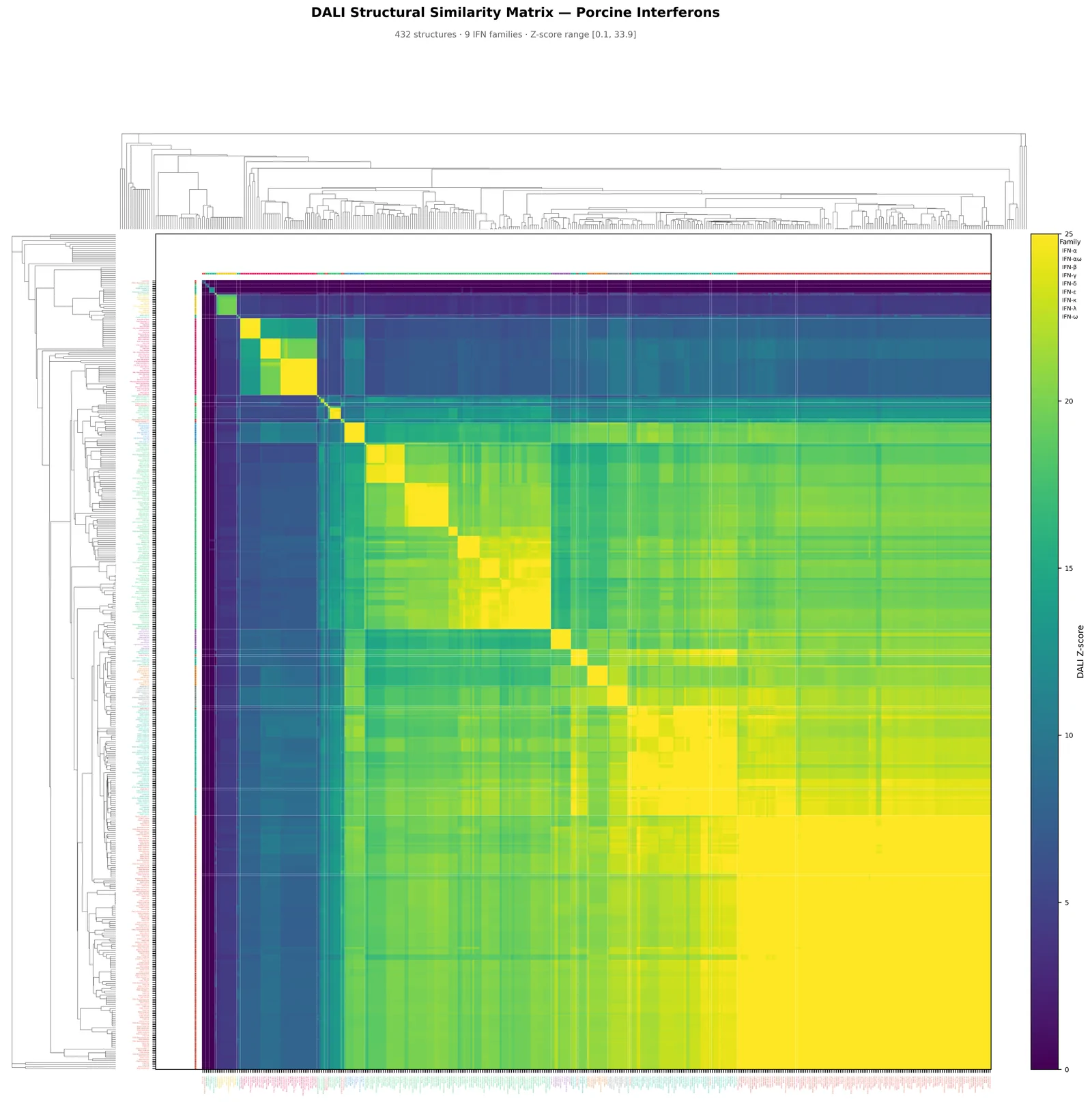
DALI structural similarity matrix of porcine IFN proteins. Hierarchically clustered heatmap of pairwise DALI Z-scores computed from all-against-all structural comparisons of 432 predicted IFN protein structures spanning 9 IFN families. Color scale ranges from dark purple (Z-score ≈ 0, no structural similarity) to bright yellow (Z-score up to 33.9, high structural similarity). Row and column color bars indicate IFN family identity: IFN-α (red), IFN-β (orange), IFN-γ (yellow), IFN-δ (green), IFN-ε (light blue), IFN-κ (dark blue), IFN-λ (pink), and IFN-ω (teal). Dendrograms on both axes represent hierarchical clustering based on structural similarity. Distinct diagonal blocks of high Z-scores reflect strong intra-family structural conservation, while off-diagonal gradients indicate varying degrees of inter-family structural relatedness. A Z-score threshold of 2.0 is conventionally used to denote statistically significant structural similarity.

**Figure 8:**
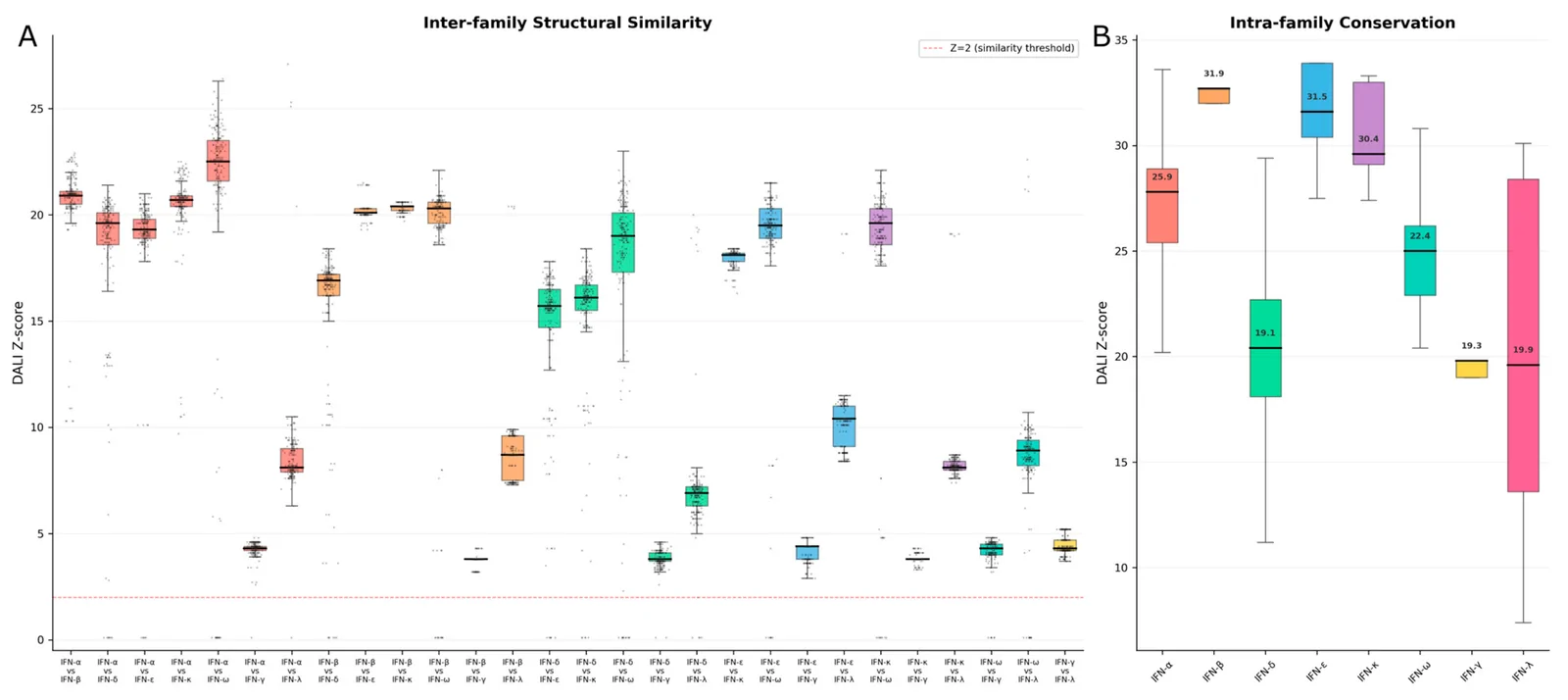
Inter- and intra-family structural similarity of porcine IFN proteins based on DALI Z-scores. (**A**) Box plots showing the distribution of pairwise DALI Z-scores for all inter-family comparisons across the nine porcine IFN subfamilies. Each box represents a pairwise family combination, color-coded by the comparison group. The red dashed horizontal line marks the Z = 2 significance threshold for structural similarity. IFN-α versus IFN- IFN-ω comparisons yielded the highest inter-family Z-scores, indicating the greatest cross-family structural relatedness, while comparisons involving IFN-δ with distantly related families produced the lowest scores. (**B**) Box plots of intra-family DALI Z-scores summarizing structural conservation within each IFN family. Median Z-scores are annotated above each box. IFN-β exhibited the highest intra-family structural conservation (median Z = 31.9), followed by IFN-αω (31.2) and IFN-κ (30.4), while IFN-γ (19.3) and IFN-δ (19.1) showed the lowest intra-family conservation, suggesting greater structural diversity among paralogs within these families.

The 432 × 432 DALI Z-score matrix ordered by average-linkage clustering revealed a clear block-diagonal structure in which sequences grouped by IFN subtypes rather than by breed of origin (Figure 7). The largest and most internally diverse block corresponded to IFN-α (*n* = 147), followed by IFN-δ (*n* = 116) and IFN-ω (*n* = 71). A visually distinct low-similarity region was apparent along the rows and columns corresponding to IFN-γ, which showed consistently low Z-scores against all other families, appearing as a dark band across the matrix. IFN-λ occupied an intermediate position, with moderate similarity to Type I families and low similarity to IFN-Intra-family mean Z-scores varied across the nine subfamilies (Figure 8).

The highest intra-family structural conservation was observed in IFN-β (mean Z = 31.9), IFN-ε (mean Z = 31.5), IFN-αω (mean Z = 31.2), and IFN-κ (mean Z = 30.4), followed by IFN-α (mean Z = 25.9), IFN-ω (mean Z = 22.4), IFN-δ (mean Z = 19.1), IFN-λ (mean Z = 19.9), and IFN-γ (mean Z = 19.3). Inter-family Z-scores differed substantially across family pairs (Figure 8A). Among Type I IFN family comparisons, IFN-α versus IFN-ω showed the highest inter-family similarity (mean Z ≈ 22), followed by IFN-α versus IFN-αω, IFN-α versus IFN-β, IFN-α versus IFN-ε, and IFN-α versus IFN-κ, all with mean Z-scores exceeding 20. IFN-β, IFN-ε, IFN-κ, IFN-γ and IFN-αω pairwise inter-family comparisons likewise yielded mean Z-scores near 20, consistent with their shared single-gene Type I IFN identity. IFN-δ showed somewhat lower inter-family similarity to other Type I IFN members, with mean Z-scores ranging from approximately 8 (IFN-δ versus IFN-λ) to 17 (IFN-δ versus IFN-α). All Type I IFNs versus Type II IFN-γ comparisons yielded mean Z-scores near 4, consistently close to the Z = 2 significance threshold, while IFN-λ versus IFN-γ comparisons similarly produced low mean Z-scores near 4. Type III IFN-λ versus Type I IFNs comparisons yielded intermediate mean Z-scores near 7–9. All inter-family comparisons involving IFN-γ remained well below the mean Z-scores observed for any Type I IFN subtype pair, confirming the structural isolation of IFN-γ from the broader interferon fold shared by Type I and Type III IFN families.

To examine structural relationships at the broader IFN type level, all 432 structures were grouped into Type I (*n* = 378), Type II (*n* = 11), and Type III (*n* = 43) IFNs (Figure S3). Mean within-type DALI Z-scores were 20.0, 19.3, and 19.9 for Type I, Type II, and Type III IFNs, respectively. The broader distribution observed for Type I reflected its inclusion of seven structurally distinct families. Inter-type comparisons revealed a clear hierarchy: Type I and Type III IFNs showed the greatest similarity, with a mean Z-score of 8.2, whereas Type I–Type II and Type II–Type III comparisons showed substantially lower mean Z-scores of 4.0 and 4.5, respectively. Thus, IFN-λ was structurally more similar to Type I IFNs than to IFN-γ, while IFN-γ remained the most structurally distinct IFN type.

**Figure 9:**
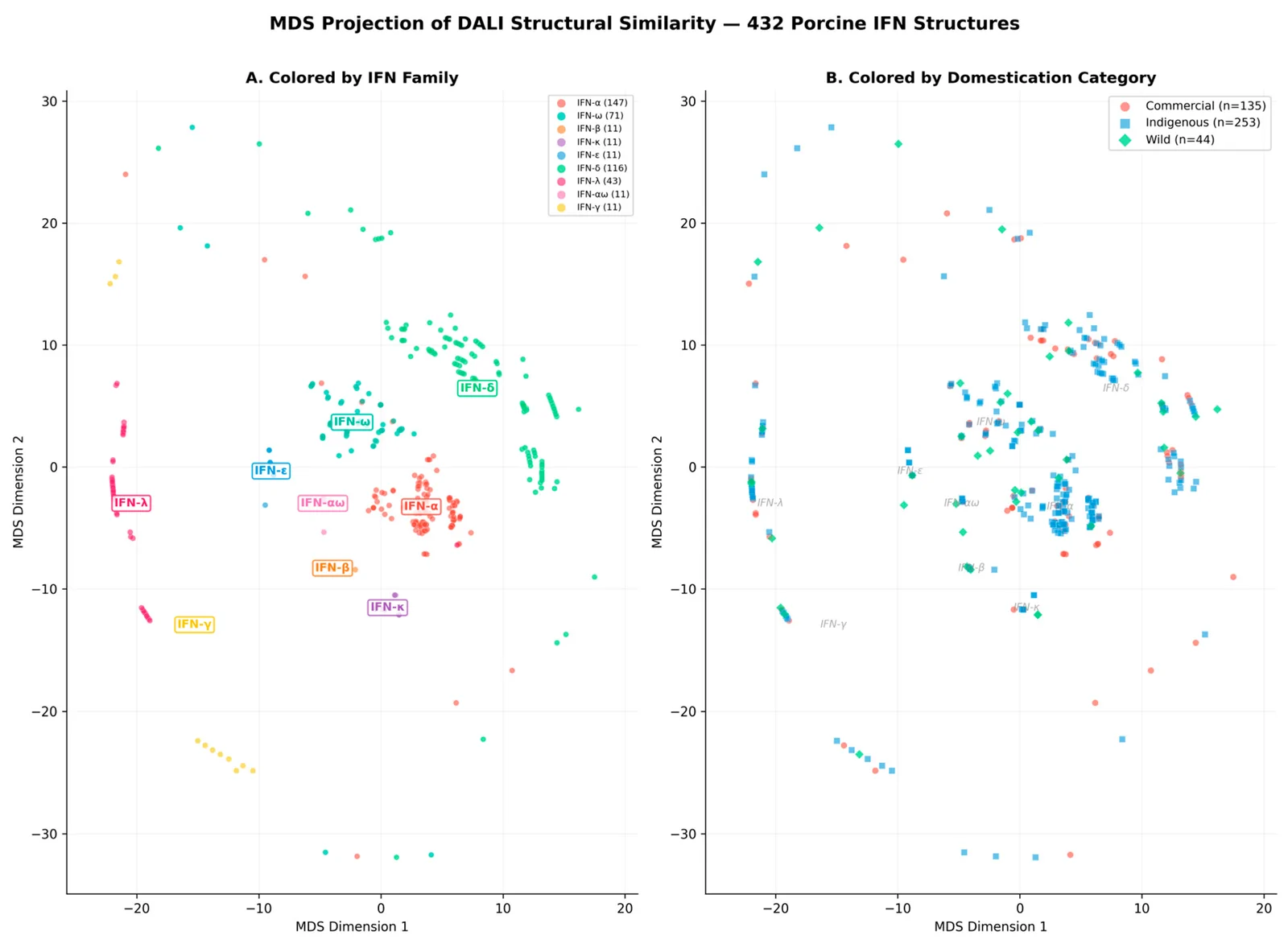
Multidimensional scaling (MDS) projection of DALI structural similarity scores across 432 porcine IFN protein structures. Two-dimensional MDS ordination was computed from pairwise DALI Z-scores to visualize the global structural relationships among all predicted IFN structures. (**A**) Structures colored by IFN family: IFN-α (red, *n* = 147), IFN-ω (teal, *n* = 71), IFN-β (orange, *n* = 11), IFN-κ (purple, *n* = 11), IFN-ε (light blue, *n* = 11), IFN-δ (green, *n* = 116), IFN-λ (pink, *n* = 43), IFN-αω (light pink, *n* = 11), and IFN-γ (yellow, *n* = 11). Structures segregate into discrete, well-separated family-level clusters, demonstrating that IFN family membership—and, by extension, functional identity—is the primary determinant of three-dimensional protein fold. (**B**) The same MDS projection with structures colored by breed domestication category: commercial (red circles, *n* = 135), indigenous (blue squares, *n* = 253), and wild (green diamonds, *n* = 44). Within each IFN family cluster, structures from commercial, indigenous, and wild breeds are thoroughly intermixed without category-level segregation, indicating that domestication history and artificial selection did not generate distinct structural variants. Taken together, panels A and B demonstrate that functional constraints imposed by IFN family membership drive structural conservation across breeds, while domestication-associated selective pressures left the IFN protein fold essentially unchanged—consistent with strong purifying selection maintaining structural integrity regardless of breed origin.

To visualize the global organization of structural relationships among all 432 IFN protein structures, pairwise DALI Z-scores were projected into two-dimensional space using multidimensional scaling (Figure 9). In Panel A, colored by IFN family, structures partitioned into discrete spatial clusters corresponding to each of the nine IFN subfamilies, with minimal overlap between family clusters. IFN-α formed the largest and most internally dispersed cluster near the center-right of the projection, flanked by IFN-ω and IFN-δ occupying adjacent but distinct regions. The IFN-αω family formed a separate compact cluster in the center-left region of the projection, spatially distinct from both the IFN-α and IFN-ω clusters. IFN-β, IFN-ε, and IFN-κ each formed compact, tightly grouped clusters consistent with their high intra-family Z-scores (31.9, 31.5, and 31.2, respectively) reported in Section 3.5. IFN-λ occupied a spatially isolated position on the left side of the projection, while IFN-γ formed a separate compact cluster in the lower-left quadrant, distant from all Type I IFN family clusters. Several outlier points were visible at the periphery of the projection, corresponding to the truncated structures identified in Section 3.4 (IFNA16-Nero Siciliano, IFNA15-Nanchukmacdon, and IFNW8 variants).

In Panel B, the same MDS coordinates are displayed with points colored by domestication category (commercial, *n* = 135; indigenous, *n* = 253; wild, *n* = 44). Across all IFN family clusters, commercial, indigenous, and wild breed sequences were spatially interleaved without forming category-specific sub-clusters. Within the IFN-α, IFN-δ, and IFN-ω clusters—the three largest and most internally variable family groups—points from all three domestication categories occupied overlapping regions of structural similarity space. This pattern was consistent across all nine IFN subfamilies, with no family cluster showing spatial segregation by domestication category.

### 3.6. Structural Conservation and Sequence-Level Selection Are Evolutionarily Decoupled

**Figure 10:**
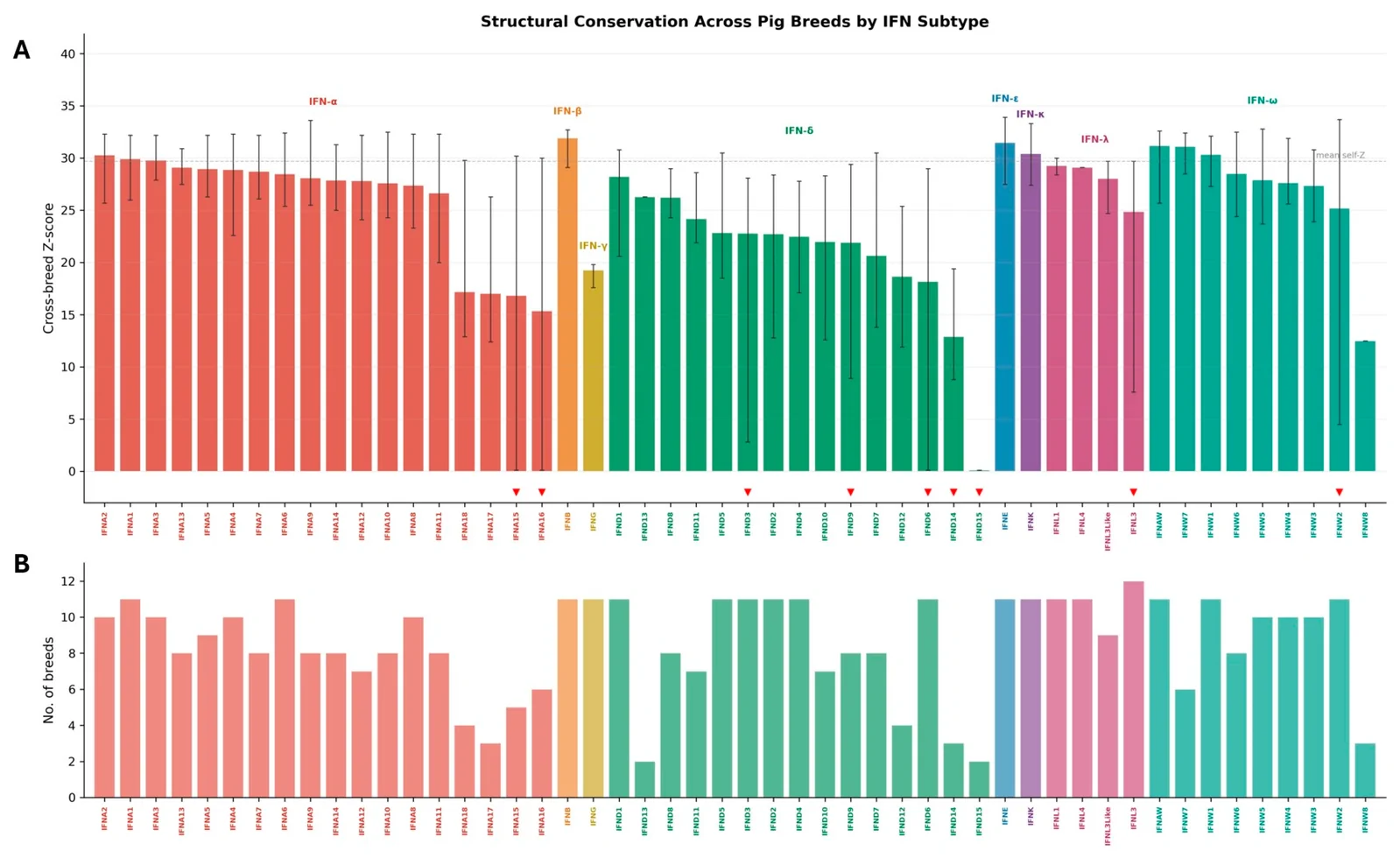
Subtype-level structural conservation of porcine IFN proteins across breeds. (**A**) Mean cross-breed DALI Z-scores for each of the 50 IFN subtypes represented in at least two breeds, with error bars representing the minimum-to-maximum range across all breed-pair comparisons. Subtypes are grouped and color-coded by IFN family: IFN-α (red), IFN-β (orange), IFN-γ (gold), IFN-δ (green), IFN-ε (blue), IFN-κ (purple), IFN-λ (dark pink), IFN-ω (teal), and IFN-αω (bright pink). The gray dashed horizontal line indicates the mean self-Z reference. Red triangles below the x-axis mark subtypes containing at least one breed pair with a minimum Z-score < 10, corresponding to truncated or structurally divergent sequences. Single-copy families (IFN-β, IFN-ε, IFN-κ, IFN-γ, IFN-αω) exhibited the highest and most consistent cross-breed conservation (mean Z = 19–32). Among multi-copy families, most IFN-α and IFN-ω subtypes showed high conservation (mean Z ≈ 25–31), whereas IFNA15–18 and IFNW8 were notably lower with wider variance. IFN-δ displayed the broadest range of cross-breed structural conservation across subtypes (mean Z ≈ 13–28). (**B**) Number of breeds represented per IFN subtype, reflecting variable subtype presence across the 11 breeds included in this study.

To quantify structural conservation at the subtype level across all 11 breeds, mean cross-breed DALI Z-scores were computed for each of the 50 IFN subtypes represented in at least two breeds and displayed alongside the minimum-to-maximum range (Figure 10A). The number of breeds represented per subtype is shown in Figure 10B.

Single-copy families showed the highest and most consistent cross-breed structural conservation. IFN-β, IFN-ε, IFN-αω, IFN-γ, and IFN-κ each displayed mean cross-breed Z-scores between 25 and 32, with narrow error bars indicating minimal inter-breed structural variation. IFN-γ showed similarly high mean Z-scores near 19–20 with very low variance, consistent with its single-copy status and compact intra-family clustering observed in Section 3.5.

Among multi-gene Type I IFN families, IFN-α subtypes maintained high cross-breed conservation, with mean Z-scores ranging from approximately 27 to 30 across all subtypes except IFNA15, IFNA16, IFNA17, and IFNA18, which showed lower mean Z-scores (approximately 15–17) and wider error bars. These lower-conservation subtypes were flagged by red triangles in Figure 10A, indicating the presence of at least one breed pair with minimum Z < 10, corresponding to the truncated structures identified in Section 3.4.

IFN-δ displayed the widest range of cross-breed conservation among all families. Mean Z-scores across IFN-δ subtypes ranged from approximately 13 (IFND15) to 28 (IFND1), with subtypes IFND6, IFND14, and IFND15 showing the lowest mean values and the broadest error bars. Multiple IFN-δ subtypes were flagged with red triangles, indicating breed pairs with structurally divergent comparisons. IFN-ω subtypes were generally well-conserved, with mean Z-scores between 25 and 31 across most subtypes, with the exception of IFNW8 (mean Z ≈ 13), which showed poor cross-breed conservation and was flagged accordingly. IFN-λ subtypes showed intermediate conservation, presenting mean Z-scores ranging from approximately 25 to 29 across IFNL1, IFNL3, IFNL3-like, and IFNL4, with IFNL3 displaying somewhat wider variance than the other λ subtypes.

**Figure 11:**
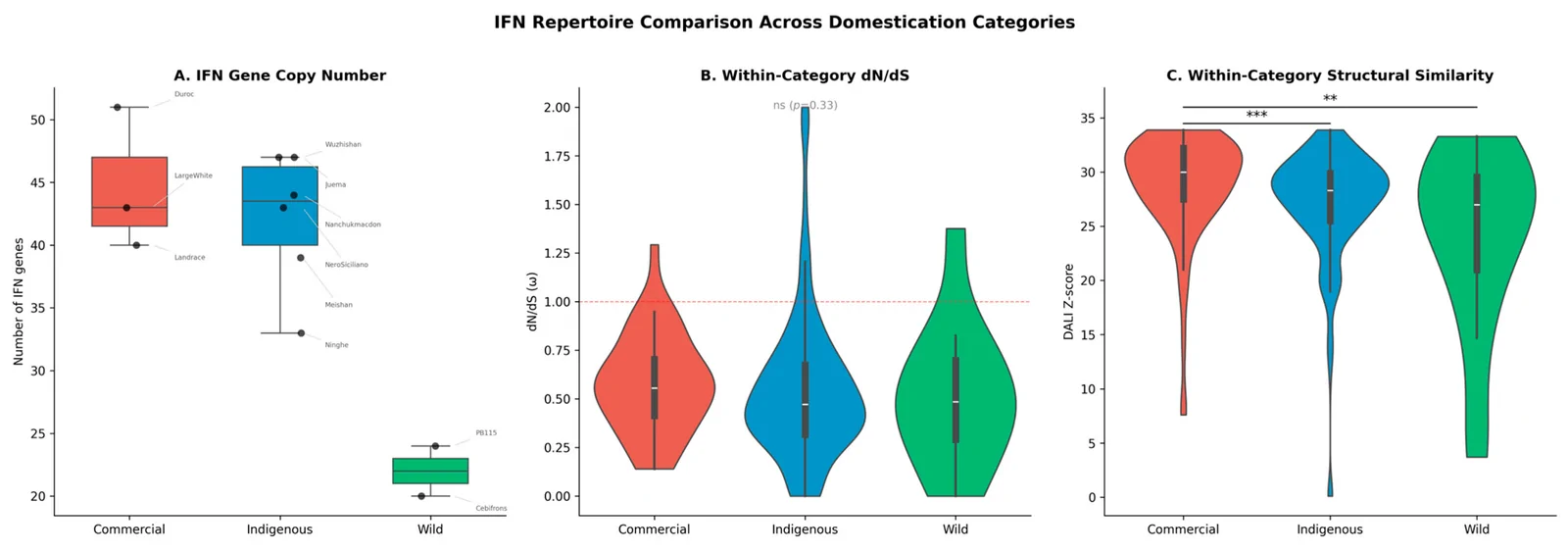
Comparison of IFN repertoire size, selective pressure, and structural similarity across domestication categories. Breeds were classified into commercial (*n* = 3; Large White, Landrace, Duroc), indigenous (*n* = 6; Wuzhishan, Juema, Nanchukmacdon, Nero Siciliano, Meishan, Ninghe), and wild (*n* = 2; PB115/Bushpig, *Sus cebifrons*) categories. Groups were compared using Kruskal–Wallis tests followed by post hoc pairwise Mann–Whitney U tests (two-sided, uncorrected). (**A**) Box plots of total IFN gene copy number per breed. Commercial and indigenous breeds showed comparable median repertoire sizes (median = 43 and 43.5, respectively), while wild taxa harbored markedly smaller repertoires (mean = 22.0). Repertoire differences were driven exclusively by multi-copy families (IFN-α, IFN-δ, IFN-ω); single-copy families were invariant across all categories. The overall difference did not reach statistical significance (Kruskal–Wallis, H = 4.644, *p* = 0.098). (**B**) Violin plots of within-category pairwise dN/dS (ω) distributions. Median ω values were 0.555 (commercial), 0.472 (indigenous), and 0.484 (wild), all well below the neutral threshold of 1.0 (red dashed line). No significant difference was detected across or between any category pair (Kruskal–Wallis, H = 2.192, *p* = 0.334; all post hoc *p* > 0.05), indicating domestication did not alter the selective regime acting on IFN-coding sequences. (**C**) Violin plots of within-category pairwise DALI Z-scores for orthologous IFN structure pairs. Structural similarity differed significantly across categories (Kruskal–Wallis, H = 23.939, *p* = 6.33 × 10^−6^). Commercial breeds showed the highest median intra-category Z-score (median = 30.0), followed by indigenous (median = 28.3) and wild lineages (median = 27.0). Post hoc comparisons revealed significant differences between commercial and indigenous breeds (Mann–Whitney U, *p* = 4.01 × 10^−6^, ***) and between commercial and wild taxa (*p* = 5.34 × 10^−3^, **); the indigenous versus wild comparison was not significant (*p* = 0.158).

To examine whether domestication history is associated with differences in IFN gene copy number, sequence evolution, or structural similarity, breeds were classified into three categories—commercial (*n* = 3), indigenous (*n* = 6), and wild/outgroup (*n* = 2)—and three metrics were compared across groups using Kruskal–Wallis tests followed by post hoc pairwise Mann–Whitney U tests (two-sided, uncorrected) (Figure 11).

IFN gene copy number differed across domestication categories, though the difference did not reach statistical significance (Kruskal–Wallis, H = 4.644, *p* = 0.098) (Figure 11A). Commercial breeds showed a mean of 45 genes per breed (median = 43, range 40–51), and indigenous breeds showed a similar mean of 42.2 (median = 43.5, range 33–47) but with substantially wider variance across the six breeds. Wild lineages showed consistently smaller repertoires, with PB115 (*n* = 24) and *Sus cebifrons* (*n* = 20) both falling well below all domestic breeds (mean = 22.0). Per-family breakdown confirmed that repertoire differences were driven exclusively by multi-gene IFN subfamilies: IFN-α (commercial 17.0 ± 1.6, indigenous 16.0 ± 4.0, wild 5.5 ± 2.5), IFN-δ (12.0 ± 2.2, 11.3 ± 1.2, 6.0 ± 0.0), and IFN-ω (7.7 ± 0.9, 6.8 ± 0.9, 3.5 ± 0.5), while all single-gene IFN families (IFN-β, -γ, -ε, -κ, -αω) showed identical counts of 1.0 ± 0.0 across all three categories.

Within-category pairwise dN/dS values were broadly similar across all three categories (Kruskal–Wallis, H = 2.192, *p* = 0.334) (Figure 11B). Median ω values were 0.555 for commercial (*n* = 35 pairs), 0.472 for indigenous (*n* = 370 pairs), and 0.484 for wild (*n* = 12 pairs). No post hoc pairwise comparison reached significance (Commercial vs. Indigenous: *p* = 0.146; Commercial vs. Wild: *p* = 0.323; Indigenous vs. Wild: *p* = 0.872). All three distributions showed the majority of pairs below ω = 1. To further examine whether sequence-level selective pressure and structural similarity are coupled, pairwise DALI Z-scores were matched to corresponding dN/dS values for 83,028 structure–sequence pairs (Figure S4). Linear regression yielded R^2^ < 0.001 with a slope of −0.0003 across all pairs and R^2^ < 0.001 (*p* = 0.607) for the 1056 ortholog pairs independently, indicating no meaningful association between structural similarity and selective pressure across any comparison class.

Within-category structural similarity Z-scores differed significantly across domestication categories (Kruskal–Wallis, H = 23.939, *p* = 6.33 × 10^−6^) (Figure 11C). Commercial breeds showed the highest median intra-category ortholog Z-score (median = 30.0, mean = 28.7, *n* = 125 pairs), followed by indigenous breeds (median = 28.3, mean = 27.0, *n* = 547 pairs) and wild/outgroup lineages (median = 27.0, mean = 23.9, *n* = 18 pairs). Post hoc pairwise comparisons revealed a significant difference between commercial and indigenous categories (Mann–Whitney U, U = 43,216, *p* = 4.01 × 10^−6^) and between commercial and wild/outgroup categories (U = 1583, *p* = 5.34 × 10^−3^), while the difference between indigenous and wild/outgroup categories did not reach significance (U = 5884.5, *p* = 0.158).

## 4. Discussion

### 4.1. Expansion of Multi-Gene Type I IFN Families Reflects Multifactorial Evolutionary Pressures

The present study provides a comprehensive cross-breed comparative analysis of the porcine IFN gene repertoire, revealing that the structural and evolutionary landscape of swine IFNs is fundamentally organized around IFN family identity rather than breed origin. Our characterization of 432 IFN sequences across 11 breeds (Figure 1), spanning three continents, confirms and extends prior work, demonstrating that the porcine Type I IFN loci have undergone remarkable expansion relative to other mammalian species, with porcine type I IFN genes undergoing active diversification through gene duplication and conversion and IFN-α, IFN-ω, and IFN-δ subfamilies showing substantial gene expansion under purifying selection [17,30]. The total repertoire size we observed across domestic breeds (range 39–51 genes) is consistent with the expanded IFN gene composition previously documented in swine compared with humans and mice and comparable to that reported in cattle, where the IFNW family is greatly expanded, comprising 24 potentially functional genes, underscoring that ungulates as a group have evolved unusually complex Type I IFN repertoires [31]. The invariance of single-copy families (IFN-β, -γ, -ε, -κ, -αω) across all 11 breeds, including wild-proxy lineages, confirms that the structural backbone of the IFN system is ancient and resistant to gene number variation, while the multi-gene subfamilies represent the primary substrate for evolutionary diversification [4]. Our breed-level data provide direct comparative evidence consistent with this hypothesis: domestic breeds consistently harbor larger IFN-α, IFN-δ, and IFN-ω repertoires than wild-proxy lineages, suggesting that repertoire expansion is at least partially associated with the transition to dense, high-contact husbandry conditions that intensify pathogen transmission pressure [17,32,33,34].

However, the specific evolutionary forces responsible for the expansion of the porcine IFN repertoire remain unresolved. Although PRRSV and African swine fever virus are important contemporary swine pathogens, their documented immune-evasion mechanisms predominantly interfere with IFN induction or intracellular signaling rather than directly binding and neutralizing secreted IFN ligands. PRRSV nsp1β, for example, disrupts nuclear translocation of the STAT1/STAT2/IRF9 complex, whereas several ASFV proteins target components of the cGAS–STING, TBK1, and JAK–STAT pathways [35,36,37]. These interactions demonstrate the central importance of the IFN system during infection but do not establish that these particular viruses directly drove the expansion of IFN ligand genes. Swinepox virus provides a potentially more direct host–virus interaction relevant to IFN ligand diversification. Its genome encodes SPV132, a homolog of poxviral IFN-α/β-binding proteins, as well as SPV008, a homolog of viral IFN-γ receptor proteins [38,39]. A viral IFN-binding protein could theoretically favor host IFN variants that maintain cellular receptor activation while reducing sequestration by the viral decoy. Thus, swinepox virus or related poxviruses may have contributed to the long-term selective environment affecting porcine IFNs. However, SPV132 was identified through comparative genome annotation, and its binding specificity toward individual porcine IFN subtypes and its evolutionary effects on host IFN loci have not been directly established. Swinepox virus should therefore be regarded as a plausible candidate rather than a demonstrated primary driver.

More generally, the porcine IFN repertoire likely reflects the combined effects of recurrent exposure to multiple pathogens, gene duplication and conversion, genomic architecture, lineage-specific retention, dosage effects, and functional specialization. Previous analyses indicate that the expanded porcine IFN-α, IFN-δ, and IFN-ω families arose through duplication and gene conversion while remaining predominantly under purifying selection [30]. These multi-gene families are also associated with repetitive genomic elements, and their members exhibit differences in expression and antiviral activity [30]. Such functional differences may have favored the retention of particular paralogs. Accordingly, the present study does not attribute porcine IFN expansion to a single pathogen but instead supports a multifactorial model involving long-term host–pathogen interactions and lineage-specific gene-family evolution.

### 4.2. Purifying Selection Is the Predominant Constraint on IFN Coding Sequences

Pairwise dN/dS analysis across 77,004 finite sequence pairs revealed a global median ω of 0.48, with the overwhelming majority of pairs falling below the neutral threshold of ω = 1 (Figure 4). This pervasive purifying selection is consistent with porcine type I IFN genes undergoing gene expansion under purifying selection and aligns with the broader principle that helical cytokines are highly permissive to sequence perturbation in their three-dimensional fold, yet are functionally constrained at the level of receptor-binding interfaces and signaling motifs [30]. The robustness of the pairwise dN/dS estimates was supported by cross-validation using the Yang–Nielsen method implemented in yn00 from PAML4, which showed strong concordance with the Nei–Gojobori estimates used in the main analysis (Figure S1). In addition, sliding-window dN/dS analysis of representative IFN orthologs, including Type I IFNs IFN-α (IFNA1), IFN-β (IFNB), IFN-δ (IFND3), IFN-κ (IFNK), and IFN-ω (IFNW1), and Type II IFN-γ (IFNG), showed that all informative codon windows remained below ω = 1 (Figure S2). Importantly, IFN gene-repertoire size and dN/dS describe distinct levels of evolutionary change. Repertoire size is shaped by gene duplication, deletion, pseudogenization, and lineage-specific retention, whereas dN/dS measures selective constraint on the coding sequences of retained genes that can be reliably aligned and compared. Consequently, a lineage may retain a comparatively compact IFN repertoire while the coding sequences of its remaining IFN genes remain strongly conserved by purifying selection. This distinction is consistent with comparative studies showing that the origin, retention, and divergence of duplicated genes do not necessarily proceed in parallel with the selective constraints acting on established coding sequences [40]. Therefore, the smaller recovered IFN repertoire in the two wild/outgroup reference lineages does not conflict with the predominantly low dN/dS ratios observed across the sampled IFN genes.

Taken together, our findings support a two-level evolutionary model in which IFN copy number changes through lineage-specific duplication, loss, pseudogenization, or retention, while essential receptor-binding and signaling functions remain constrained at the coding-sequence level. These results further support the predominance of purifying selection across both global pairwise comparisons and localized coding regions. The observed pattern—abundant sequence variation accommodated within a conserved fold framework—is precisely what would be expected if natural selection acts primarily to preserve receptor recognition and signaling competence rather than to maintain any specific amino acid sequence per se [41].

Furthermore, the phylogenetic analysis demonstrated that IFN paralogs cluster by subtype identity rather than breed of origin (Figure 3), indicating that the diversification of numbered subtypes predates breed divergence by a substantial evolutionary interval. This finding has an important implication: the IFN subtype repertoire is not a plastic trait that breeds have independently modified but rather an ancestral framework that was largely established before domestication began and has been maintained under purifying selection ever since [42,43]. The small number of breed-specific subtypes (IFNA19, IFNA20, IFNA21, IFND13, IFND15, IFNW9, each represented in a single breed) represent exceptions that may reflect either recent lineage-specific duplications or incomplete detection in other assemblies and warrant further investigation. The between-breed ortholog heatmap (Figure 5) further reinforces this picture: no breed pair exceeded a median ω of 1.0, and the elevated values observed for Ninghe–*Sus cebifrons* (ω = 0.76) and Meishan–*Sus cebifrons* (ω = 0.68) reflect the greater evolutionary distance between Chinese indigenous breeds and the wild-proxy outgroup rather than any departure from purifying selection as the dominant regime.

### 4.3. Structural Conservation Is Governed by Family Identity, Not Breed Origin

The DALI-based structural similarity analysis of 432 AlphaFold2-predicted structures revealed a clear and consistent pattern: pairwise Z-scores partition by IFN family, with all Type I IFN subfamilies forming a structurally coherent supercluster while IFN-γ remains structurally isolated from all other families (Figure 7, Figure 8 and Figure 9). Comparative analysis of IFN-γ across vertebrates reveals an overall conservation of the fold, dimer interface, and some interactions with the receptor despite very low sequence identities, which is fully consistent with our finding that IFN-γ sequences across 11 swine breeds cluster tightly in both sequence-based phylogeny and structural MDS space despite showing the lowest inter-family Z-scores against all Type I IFNs [44]. At the broader IFN type level, the structural relationships observed in Figure S3 also provide context for the distinct receptor systems used by Type I, Type II, and Type III IFNs. Although Type I and Type III IFNs signal through different receptor complexes, their higher inter-type structural similarity is consistent with their overlapping antiviral transcriptional programs. Their distinct receptor utilization is therefore likely determined not solely by the global protein fold but also by differences in surface residues and receptor-interaction geometry. In contrast, the marked structural separation of IFN-γ is consistent with its distinct receptor complex and its functional emphasis on cell-mediated immune regulation. These associations should be interpreted cautiously because global DALI similarity does not directly measure receptor binding or functional activity [44].

The decoupling of structural similarity from sequence-level selective pressure (R^2^ < 0.001, Figure S4) confirms that these two evolutionary signals are genuinely independent. The three-dimensional fold of helical cytokines is particularly permissive to sequence perturbation, which explains why orthologous IFN pairs with Z-scores near 30—essentially identical structures—can still show dN/dS values distributed across the full range from 0.1 to above 1.0. Structural conservation is maintained by the thermodynamic stability of the four-helix bundle topology, while sequence conservation is maintained by functional selection at receptor-contacting residues. These are complementary but mechanistically distinct constraints [45].

The MDS projection of structural similarity space (Figure 9B) provided the clearest visual evidence that domestication category does not predict structural variant identity: commercial, indigenous, and wild breed sequences are thoroughly intermixed within every IFN family cluster, with no spatial separation by breeding history. This finding demonstrates that domestication did not generate new structural IFN variants. Whatever diversification occurred in the gene number or sequence during breed formation, it did not alter the fundamental structural identity of any IFN subfamily [46].

### 4.4. Domestication Drove IFN Gene Expansion and Structural Convergence While Preserving Selective Constraint

The domestication comparison analysis reveals a coherent and directional immunogenetic signature that deepens progressively from wild/outgroup lineages through indigenous breeds to commercial breeds. Three converging lines of evidence support this conclusion.

First, the functional IFN gene number is substantially lower in wild-proxy lineages (mean = 22.0) than in domestic breeds (mean = 42–45), consistent with the co-evolution of the porcine IFN complex with increasing viral pressure after domestication [17]. Although this difference did not reach statistical significance (Kruskal–Wallis *p* = 0.098), the biological magnitude is striking—domestic breeds carry on average twice as many IFN genes as wild-proxy lineages—and the trend is entirely driven by the multi-gene Type I subfamilies including IFN-α, IFN-δ, and IFN-ω subtypes, the same subtypes that show the greatest breed-level variability throughout this study. The lack of statistical significance most likely reflects the small sample size of wild-proxy lineages (*n* = 2) rather than the absence of a true biological signal. Importantly, a smaller recovered IFN repertoire should not be interpreted as evidence of weaker immunity. Broader pathogen exposure in wild populations does not necessarily require a larger number of IFN genes because IFNs are broadly acting cytokines rather than pathogen-specific recognition molecules. Antiviral capacity also depends on inducible expression, cell- and tissue-specific cytokine production, receptor abundance, signaling kinetics, downstream interferon-stimulated genes, and interactions with other innate and adaptive immune pathways. Thus, IFN copy number represents only one component of antiviral immunity and cannot be used as a direct measure of overall immune strength or pathogen resistance.

Comparative immunophenotyping further supports a more complex distinction between wild boar and domestic pigs than a simple “stronger versus weaker” immune-system model. Wild boar have been reported to exhibit higher frequencies of total T cells and γδ T cells and elevated activation markers in selected T-cell populations, but they also showed reduced frequencies of polymorphonuclear cells and natural killer cells, lower SLA-DR expression in antigen-presenting-cell populations, and fewer differentiated memory T cells [47]. These observations indicate differences in immune-cell composition and activation state rather than uniformly greater immune capacity. Experimental infection studies similarly show that immune outcomes are pathogen- and context-dependent. Following infection with a moderately virulent classical swine fever virus, wild boar and domestic pigs were both susceptible and carried broadly comparable viral loads, although some clinical and immunological parameters differed [48]. In a moderately virulent African swine fever virus model, wild boar showed greater viral-antigen loads in several tissues and more persistent disease than domestic pigs [49]. These findings caution against assuming that greater environmental pathogen exposure or activation of selected immune populations necessarily results in superior resistance.

Second, within-category dN/dS ratios are statistically indistinguishable across commercial, indigenous, and wild categories (Kruskal–Wallis *p* = 0.334). Despite the dramatic differences in breeding history, population size, and pathogen exposure among these groups, the selective pressure acting on IFN coding sequences has remained constant across the entire domestication spectrum. Domestication added genes to the repertoire—sometimes doubling the count relative to wild ancestors—without relaxing or intensifying the evolutionary constraint on those genes. This result is not contradictory to the observed difference in gene number because repertoire size and dN/dS measure different evolutionary processes. IFN repertoire size is shaped by duplication, deletion, pseudogenization, and lineage-specific retention, whereas dN/dS measures selective constraint on the coding sequences of genes that remain and can be reliably compared. Consequently, a lineage may retain a relatively compact IFN repertoire while its remaining IFN coding sequences remain strongly conserved by purifying selection. Comparative studies of duplicated genes similarly demonstrate that gene-duplication history and coding-sequence constraint need not change in parallel [43,50]. The IFN system appears to follow the same principle: domestication reshapes repertoire architecture without fundamentally altering the codon-level constraint that preserves IFN function.

Third, and most strikingly, within-category structural similarity Z-scores were significantly higher in commercial breeds than in either indigenous breeds (*p* = 4.01 × 10^−6^) or wild lineages (*p* = 5.34 × 10^−3^), while the difference between indigenous and wild categories was not significant (*p* = 0.158). This result is not merely a statistical contrast—it is a direct genomic signature of domestication at work. The progressive increase in structural similarity from wild lineages through indigenous breeds to commercial breeds mirrors precisely the trajectory of IFN gene number expansion, where wild-proxy lineages consistently carried the smallest repertoires and commercial breeds the largest. Together, these two lines of evidence tell a coherent story: domestication drove IFN gene expansion, and that expansion was accompanied by increasing structural convergence among the duplicated paralogs. The more IFN genes a breed carries as a consequence of the sustained pathogen pressure and selective environment of domestic husbandry, the more structurally uniform those genes become within the breed, as gene duplication events preferentially produce copies that closely resemble their parental template. Generation after generation of domestication and artificial selection have compounded this effect, such that commercial breeds now show both the largest IFN repertoires and the most structurally homogeneous IFN proteins. The significant structural similarity differences we observe between domestication categories therefore directly reflect the depth and intensity of the domestication process itself—wild breeds retain structural diversity, while the most intensively domesticated commercial breeds have converged toward a structurally narrower but numerically expanded repertoire. This convergence of copy number expansion and structural homogenization under domestication represents a previously undescribed dimension of immunogenetic change during livestock breed formation.

The functional implications of this domestication-driven pattern are significant. Given that porcine type I IFNs show diverse and subtype-specific antiviral activities against major swine pathogens including PRRSV and African swine fever virus [30,51,52], the expansion of the IFN repertoire in domestic breeds likely broadens the antiviral response capacity available under pathogen challenge. At the same time, the structural convergence observed in commercial breeds raises the question of whether the expanded but homogenized repertoire offers equivalent functional breadth to the smaller but more structurally diverse repertoires retained by indigenous breeds and wild lineages. Moreover, increased or prolonged IFN activity is not invariably beneficial, as sustained Type I IFN signaling can promote chronic immune activation, induce negative regulatory pathways, and disrupt lymphoid-tissue organization [53]. These findings highlight potential biological trade-offs between repertoire expansion, structural diversification, antiviral activity, and immune-mediated pathology. Indigenous breeds, which occupy an intermediate position in both gene number and structural diversity, may represent an underexplored reservoir of immunogenetic variation with relevance to disease resistance breeding programs. Functional studies integrating IFN expression, receptor engagement, downstream signaling, and pathogen-challenge experiments will be required to determine whether the observed genomic patterns translate into meaningful differences in antiviral protection.

### 4.5. Limitations and Future Directions

Several limitations of the present study should be acknowledged. First, the wild-proxy group was represented by only two scaffold-level assemblies, PB115 and *Sus cebifrons*. Based on our survey of the available genome datasets at the time of data collection, these were the only wild-related pig genomes suitable for inclusion, and no comparable chromosome-level wild pig assembly was available. This limited sample size reduces the statistical power of domestication-category comparisons and precludes generalization to the full diversity of wild *Sus scrofa* populations across Eurasia. In addition, the scaffold-level contiguity of these two assemblies may have contributed to incomplete recovery or collapse of duplicated IFN loci, potentially leading to underestimation of IFN gene copy number in the wild-proxy group. Therefore, the observed differences should be interpreted as a domestication-associated pattern within the genomes examined rather than as definitive evidence that domestication directly caused IFN gene expansion. Future studies incorporating additional high-quality and geographically diverse chromosome-level wild boar assemblies would substantially strengthen the domestication inference. An additional limitation is that breed category and phylogeographic ancestry are partially confounded in the current sampling design. The three commercial breeds are of Western genetic origin, whereas most indigenous breeds are of Asian origin; therefore, lineage background may contribute to the observed commercial–indigenous differences. At the same time, Duroc, Large White, and Landrace are now globally distributed breeds that form the genetic foundation of intensive commercial pig production in multiple regions, including Asia. Accordingly, the observed differences are interpreted as potentially associated with commercial breeding and domestication history, while a contribution from phylogeographic ancestry cannot be excluded.

Genome assembly quality varies across the 11 breeds examined, and differences in sequencing depth or chromosomal contiguity may contribute to apparent breed differences in IFN gene count, particularly for the rare single-breed subtypes (IFNA19, IFNA20, IFNA21) whose presence in only one assembly may reflect detection gaps rather than genuine breed-specific duplications. All structural predictions were generated computationally using AlphaFold2, and while mean pLDDT scores across the passing dataset were high, the accuracy of predictions for truncated or divergent sequences—precisely the outliers most relevant to functional interpretation—may be reduced relative to experimentally determined structures.

Three directions emerge directly from the findings of this study. First, the domestication-associated IFNL3-like duplication present in all nine domestic breeds but absent from both wild-proxy lineages warrants targeted functional characterization to determine whether it exhibits distinct receptor affinity, expression kinetics, or antiviral specificity relative to IFNL3. Second, the breed-specific pseudogenization of IFNE in Ninghe through two frameshift insertions requires follow-up through transcriptomic profiling and viral challenge experiments in Ninghe animals to assess whether compensatory upregulation of other Type I IFN subtypes occurs and whether this breed shows altered susceptibility to pathogens for which IFNE-mediated immunity is non-redundant. Third, integration of breed-level transcriptomic data—particularly from innate immune cells under viral challenge conditions—with the genomic framework established here is the essential next step toward validating IFN repertoire composition as a functional biomarker of antiviral capacity across swine breeds.

## 5. Conclusions

This study provides the first comprehensive comparative genomic analysis of the complete IFN gene repertoire across 11 swine breeds spanning three continents, integrating sequence-based phylogenetics, selection pressure analysis, and AlphaFold2-based structural prediction with DALI pairwise comparison. Across 432 curated IFN sequences representing all nine IFN subfamilies and 56 subtypes, IFN repertoire architecture is organized along two independent axes: family identity, which determines both phylogenetic clustering and structural similarity regardless of breed origin, and a potential domestication-associated pattern in gene number and structural homogeneity across the sampled wild-proxy, indigenous, and commercial groups.

Purifying selection is the predominant evolutionary regime governing IFN coding sequences across all families and all domestication categories, confirming that the functional essentiality of receptor-binding and signaling interfaces is maintained irrespective of how extensively the repertoire has expanded. The decoupling of structural conservation from sequence-level selective pressure, demonstrated by the absence of any linear relationship between DALI Z-scores and dN/dS ratios across 83,028 matched pairs, establishes that fold preservation and codon constraint operate through mechanistically independent pathways.

Within the genomes examined, the domestication-associated pattern was most apparent at the structural level. Commercial breeds carry the largest IFN repertoires and show the highest within-category structural similarity, while wild-proxy lineages carry the fewest genes and retain the greatest structural diversity. Together, these observations are consistent with the possibility that domestication and subsequent breed formation contributed to the expansion, retention, or structural homogenization of portions of the IFN repertoire. However, because the wild-proxy category was represented by only two scaffold-level assemblies, this pattern should be regarded as an association within the current dataset rather than definitive evidence that domestication directly caused these differences. Indigenous breeds retained a more moderate and variable pattern, potentially reflecting their intermediate breeding histories and broader genetic diversity.

The genomic framework developed here, combining breed-level repertoire annotation, evolutionary rate estimation, and proteome-scale structural comparison, provides a replicable foundation for immunogenetic characterization across livestock species and for connecting IFN genomic diversity to functional differences in antiviral immunity. Future inclusion of additional high-quality wild suid genomes will be essential for determining whether the domestication-associated trends observed here are broadly reproducible across the diversity of wild and domestic pig lineages.

## Figures and Tables

**Table 1:** Genome assemblies used for IFN repertoire analysis.

Breed	Accession Number	Contig N50	Sequencing Technology	Submitter	Assembly Date
Duroc (*Sscrofa11.1*)	GCA_000003025.6	48.2 Mb	PacBio	The Swine Genome Sequencing Consortium (SGSC)	7 February 2017
Wuzhishan	GCA_048338725.1	144.9 Mb	PacBio Sequel; Oxford Nanopore PromethION; Illumina NovaSeq; BioNano DLS	China Agricultural University	5 March 2025
NS (Nero Siciliano)	GCA_006511355.2	595 kb	Illumina HiSeq	University of Messina	9 April 2021
Norwegian Landrace	GCA_963921485.1	142.1 Mb	PromethION	NORWEGIAN UNIVERSITY OF LIFE SCIENCES	11 January 2024
Juema	GCA_040869115.1	64.4 Mb	PacBio Sequel	Northwest A&F University	26 July 2024
Large White	GCA_044906105.1	43.8 Mb	PacBio Sequel	Northwest A&F University	19 November 2024
Nanchukmacdon	GCA_031306245.1	5.9 Mb	PacBio Sequel	Konkuk University	14 September 2023
Ninghe	GCA_015776825.1	4.3 Mb	Illumina HiSeq; PacBio RSII	China Agricultural University	8 December 2020
Meishan	GCA_017957985.1	51 Mb	Illumina NovaSeq; PacBio Sequel	Chinese Academy of Agricultural Sciences	15 April 2021
PB115 (Bushpig)	GCA_019290145.1	223.5 kb	Illumina 10X genomics	China Agricultural University	20 July 2021
*Sus cebifrons* (Visayan warty pig)	GCA_905335845.1	159.8 kb	Illumina	WAGENINGEN UR	22 September 2022

## Data Availability

All swine genome assemblies used are available and updated at https://www.ncbi.nlm.nih.gov/datasets/genome/?taxon=9823 (accessed on 12 August 2026).

## Supplementary Materials

The following supporting information can be downloaded at: https://www.mdpi.com/article/10.3390/biom16081195/s1, Figure S1: Cross-validation of pairwise dN/dS estimates using yn00 program in PAML v4.10.10. Figure S2: Sliding-window dN/dS profiles of representative porcine IFN orthologs across breeds. Figure S3: Global structural similarity among Type I, Type II, and Type III porcine interferons. Figure S4: Relationship between sequence-level selective pressure and structural similarity across porcine IFN proteins.

## References

[1] Schoggins J.W. (2018). Recent advances in antiviral interferon-stimulated gene biology. F1000Research.

[2] Schoggins J.W. (2019). Interferon-Stimulated Genes: What Do They All Do?. Annu. Rev. Virol..

[3] Lazear H.M., Schoggins J.W., Diamond M.S. (2019). Shared and Distinct Functions of Type I and Type III Interferons. Immunity.

[4] Razzuoli E., Armando F., De Paolis L., Ciurkiewicz M., Amadori M. (2022). The Swine IFN System in Viral Infections: Major Advances and Translational Prospects. Pathogens.

[5] Fan W., Jiao P., Zhang H., Chen T., Zhou X., Qi Y., Sun L., Shang Y., Zhu H., Hu R. (2020). Inhibition of African Swine Fever Virus Replication by Porcine Type I and Type II Interferons. Front. Microbiol..

[6] Li J., Adeyemi O., Miller L.C., Sang Y. (2025). Comparative Transcriptomics Reveals Novel and Differential Long-Noncoding RNA Responses Underlying Interferon-Mediated Antiviral Regulation in Porcine Alveolar Macrophages. Pathogens.

[7] Mancera Gracia J.C., Pearce D.S., Masic A., Balasch M. (2020). Influenza A Virus in Swine: Epidemiology, Challenges and Vaccination Strategies. Front. Vet. Sci..

[8] Wang R., Zhang Y.J. (2014). Antagonizing interferon-mediated immune response by porcine reproductive and respiratory syndrome virus. BioMed Res. Int..

[9] Lunney J.K., Van Goor A., Walker K.E., Hailstock T., Franklin J., Dai C. (2021). Importance of the pig as a human biomedical model. Sci. Transl. Med..

[10] Frantz L.A.F., Bradley D.G., Larson G., Orlando L. (2020). Animal domestication in the era of ancient genomics. Nat. Rev. Genet..

[11] See M.T. (2024). Invited Review-Current status and future trends for pork production in the United States of America and Canada. Anim. Biosci..

[12] Tang S., Li D., Lu Y., Gao Z., Wang B., Liu X., Wei H., Wu J. (2026). Integrative Advances in Pig Genomics: From Reference Assemblies and Evolutionary History to the Mechanistic Dissection of Key Traits. Biology.

[13] Larson G., Burger J. (2013). A population genetics view of animal domestication. Trends Genet..

[14] La Bonnardiere C., Lefevre F., Charley B. (1994). Interferon response in pigs: Molecular and biological aspects. Vet. Immunol. Immunopathol..

[15] Ballester M., Ramayo-Caldas Y., González-Rodríguez O., Pascual M., Reixach J., Díaz M., Blanc F., López-Serrano S., Tibau J., Quintanilla R. (2020). Genetic parameters and associated genomic regions for global immunocompetence and other health-related traits in pigs. Sci. Rep..

[16] Liu L., Megens H.-J., Crooijmans R.P., Bosse M., Huang Q., van Sonsbeek L., Groenen M.A., Madsen O. (2022). The Visayan Warty Pig (Sus cebifrons) Genome Provides Insight Into Chromosome Evolution and Sensory Adaptation in Pigs. Mol. Biol. Evol..

[17] Jennings J., Sang Y. (2019). Porcine Interferon Complex and Co-Evolution with Increasing Viral Pressure after Domestication. Viruses.

[18] Mirdita M., Schütze K., Moriwaki Y., Heo L., Ovchinnikov S., Steinegger M. (2022). ColabFold: Making protein folding accessible to all. Nat. Methods.

[19] Holm L., Laiho A., Törönen P., Salgado M. (2023). DALI shines a light on remote homologs: One hundred discoveries. Protein Sci..

[20] Randolph H.E., Aracena K.A., Lin Y., Mu Z., Barreiro L.B. (2024). Shaping immunity: The influence of natural selection on population immune diversity. Immunol. Rev..

[21] Barreiro L.B., Quintana-Murci L. (2010). From evolutionary genetics to human immunology: How selection shapes host defence genes. Nat. Rev. Genet..

[22] Warr A., Affara N., Aken B., Beiki H., Bickhart D.M., Billis K., Chow W., Eory L., A Finlayson H., Flicek P. (2020). An improved pig reference genome sequence to enable pig genetics and genomics research. Gigascience.

[23] Katoh K., Standley D.M. (2013). MAFFT multiple sequence alignment software version 7: Improvements in performance and usability. Mol. Biol. Evol..

[24] Kalyaanamoorthy S., Minh B.Q., Wong T.K.F., Von Haeseler A., Jermiin L.S. (2017). ModelFinder: Fast model selection for accurate phylogenetic estimates. Nat. Methods.

[25] Wong T.K.F., Ly-Trong N., Ren H., Demotte P., Baños H., Roger A.J., Susko E., Bielow C., De Maio N., Goldman N. (2026). IQ-TREE 3: Phylogenomic Inference Software using Complex Evolutionary Models. Mol. Biol. Evol..

[26] Hoang D.T., Chernomor O., Von Haeseler A., Minh B.Q., Vinh L.S. (2018). UFBoot2: Improving the Ultrafast Bootstrap Approximation. Mol. Biol. Evol..

[27] Letunic I., Bork P. (2024). Interactive Tree of Life (iTOL) v6: Recent updates to the phylogenetic tree display and annotation tool. Nucleic Acids Res..

[28] Yang Z. (2007). PAML 4: Phylogenetic analysis by maximum likelihood. Mol. Biol. Evol..

[29] Holm L. (2020). DALI and the persistence of protein shape. Protein Sci..

[30] Sang Y., Bergkamp J., Blecha F. (2014). Molecular evolution of the porcine type I interferon family: Subtype-specific expression and antiviral activity. PLoS ONE.

[31] Walker A.M., Roberts R.M. (2009). Characterization of the bovine type I IFN locus: Rearrangements, expansions, and novel subfamilies. BMC Genom..

[32] Fröhlich E. (2024). Animals in Respiratory Research. Int. J. Mol. Sci..

[33] Landau L.J.B., Fam B.S.d.O., Yépez Y., Caldas-Garcia G.B., Pissinatti A., Falótico T., Reales G., Schüler-Faccini L., Sortica V.A., Bortolini M.C. (2021). Evolutionary analysis of the anti-viral STAT2 gene of primates and rodents: Signature of different stages of an arms race. Infect. Genet. Evol..

[34] Li J., Adeyemi O., Miller L.C., Sang Y. (2025). Comparative Transcriptomics Reveals Novel and Differential Circular RNA Responses Underlying Interferon-Mediated Antiviral Regulation in Porcine Alveolar Macrophages. Viruses.

[35] Patel D., Nan Y., Shen M., Ritthipichai K., Zhu X., Zhang Y.-J. (2010). Porcine reproductive and respiratory syndrome virus inhibits type I interferon signaling by blocking STAT1/STAT2 nuclear translocation. J. Virol..

[36] Sun M., Yu S., Ge H., Wang T., Li Y., Zhou P., Pan L., Han Y., Yang Y., Sun Y. (2022). The A137R Protein of African Swine Fever Virus Inhibits Type I Interferon Production via the Autophagy-Mediated Lysosomal Degradation of TBK1. J. Virol..

[37] Riera E., Pérez-Núñez D., García-Belmonte R., Miorin L., García-Sastre A., Revilla Y. (2021). African Swine Fever Virus Induces STAT1 and STAT2 Degradation to Counteract IFN-I Signaling. Front. Microbiol..

[38] Afonso C.L., Tulman E.R., Lu Z., Zsak L., Osorio F.A., Balinsky C., Kutish G.F., Rock D.L. (2002). The genome of swinepox virus. J. Virol..

[39] Kaiser F.K., Wiedemann A., Kühl B., Menke L., Beineke A., Baumgärtner W., Wohlsein P., Rigbers K., Becher P., Peters M. (2021). Swinepox Virus Strains Isolated from Domestic Pigs and Wild Boar in Germany Display Altered Coding Capacity in the Terminal Genome Region Encoding for Species-Specific Genes. Viruses.

[40] Kondrashov F.A., Rogozin I.B., I Wolf Y., Koonin E.V. (2002). Selection in the evolution of gene duplications. Genome Biol..

[41] Fang J., Zhang Q., Xi Y., Lang L., Wang K., Li S. (2023). Analysis of the Differential Expression and Antiviral Activity of Porcine Interferon-alpha In Vitro. Int. J. Pept. Res. Ther..

[42] Frantz L.A., Schraiber J.G., Madsen O., Megens H.-J., Cagan A., Bosse M., Paudel Y., A Crooijmans R.P.M., Larson G., Groenen M.A.M. (2015). Evidence of long-term gene flow and selection during domestication from analyses of Eurasian wild and domestic pig genomes. Nat. Genet..

[43] Groenen M.A. (2016). A decade of pig genome sequencing: A window on pig domestication and evolution. Genet. Sel. Evol..

[44] Chen S.N., Gan Z., Hou J., Yang Y.C., Huang L., Huang B., Wang S., Nie P. (2022). Identification and establishment of type IV interferon and the characterization of interferon-upsilon including its class II cytokine receptors IFN-upsilonR1 and IL-10R2. Nat. Commun..

[45] Fox B.A., Sheppard P.O., O’Hara P.J. (2009). The role of genomic data in the discovery, annotation and evolutionary interpretation of the interferon-lambda family. PLoS ONE.

[46] Zhou R., Li S., Yao W., Xie C., Chen Z., Zeng Z., Wang D., Xu K., Shen Z., Mu Y. (2021). The Meishan pig genome reveals structural variation-mediated gene expression and phenotypic divergence underlying Asian pig domestication. Mol. Ecol. Resour..

[47] Schafer A., Friedrichs V., Deutschmann P., Klein C., Blome S., Blohm U. (2026). How the wild things are: Comparative T cell phenotyping and memory T cell identification in wild boar and domestic pigs. Front. Immunol..

[48] Petrov A., Blohm U., Beer M., Pietschmann J., Blome S. (2014). Comparative analyses of host responses upon infection with moderately virulent classical swine fever virus in domestic pigs and wild boar. Virol. J..

[49] Sehl J., Pikalo J., Schäfer A., Franzke K., Pannhorst K., Elnagar A., Blohm U., Blome S., Breithaupt A. (2020). Comparative Pathology of Domestic Pigs and Wild Boar Infected with the Moderately Virulent African Swine Fever Virus Strain “Estonia 2014”. Pathogens.

[50] Gillard G.B., Grønvold L., Røsæg L.L., Holen M.M., Monsen Ø., Koop B.F., Rondeau E.B., Gundappa M.K., Mendoza J., Macqueen D.J. (2021). Comparative regulomics supports pervasive selection on gene dosage following whole genome duplication. Genome Biol..

[51] Li J., Miller L.C., Sang Y. (2024). Current Status of Vaccines for Porcine Reproductive and Respiratory Syndrome: Interferon Response, Immunological Overview, and Future Prospects. Vaccines.

[52] Renukaradhya G.J., Meng X.-J., Calvert J.G., Roof M., Lager K.M. (2015). Live porcine reproductive and respiratory syndrome virus vaccines: Current status and future direction. Vaccine.

[53] Teijaro J.R., Ng C., Lee A.M., Sullivan B.M., Sheehan K.C.F., Welch M., Schreiber R.D., De La Torre J.C., Oldstone M.B.A. (2013). Persistent LCMV infection is controlled by blockade of type I interferon signaling. Science.

