# Supplementary material for: Domestication-Driven Expansion and Structural Convergence of the Porcine Antiviral Interferon Repertoire
Source: Biomolecules. 2026 Aug 17;16(8):1195. doi: 10.3390/biom16081195 (PMC13511426; doi:10.3390/biom16081195)
Supplement: Supplementary file 1 [file biomolecules-16-01195-s001.zip › biomolecules-4471038-supplementary.pdf]

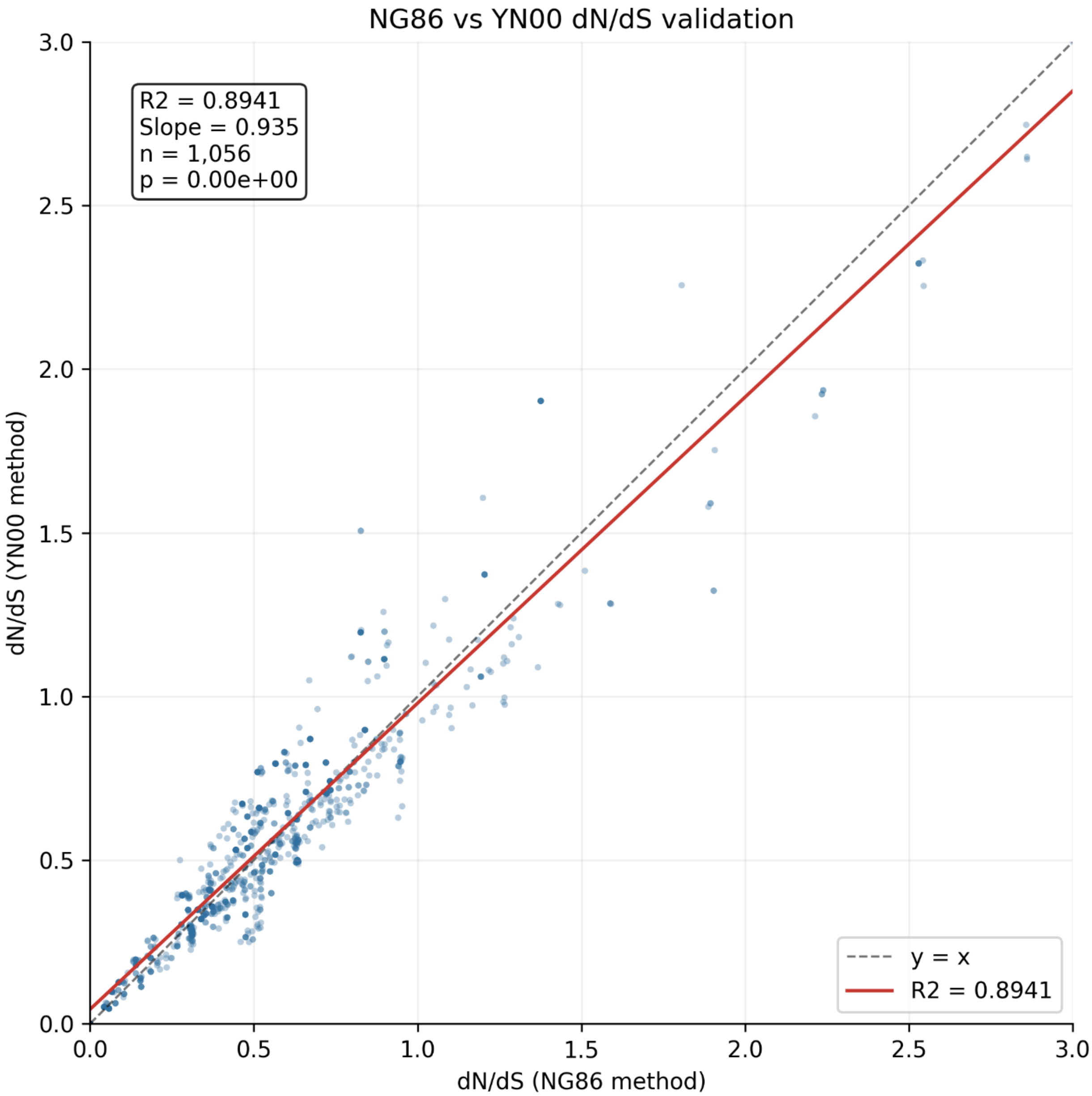

Figure S1. Cross-validation of pairwise dN/dS estimates using YN00. Scatter plot comparing pairwise dN/dS ratios estimated using the Nei-Gojobori 1986 method and the Yang-Nielsen 2000 method implemented in yn00 from PAML v4.10.10. Each point represents a matched finite same-gene sequence pair. The dashed line indicates y = x. The strong positive concordance between methods ($R^2 = 0.894$, slope = 0.935, n = 1,056) supports the robustness of the dN/dS estimates used in the main analysis.

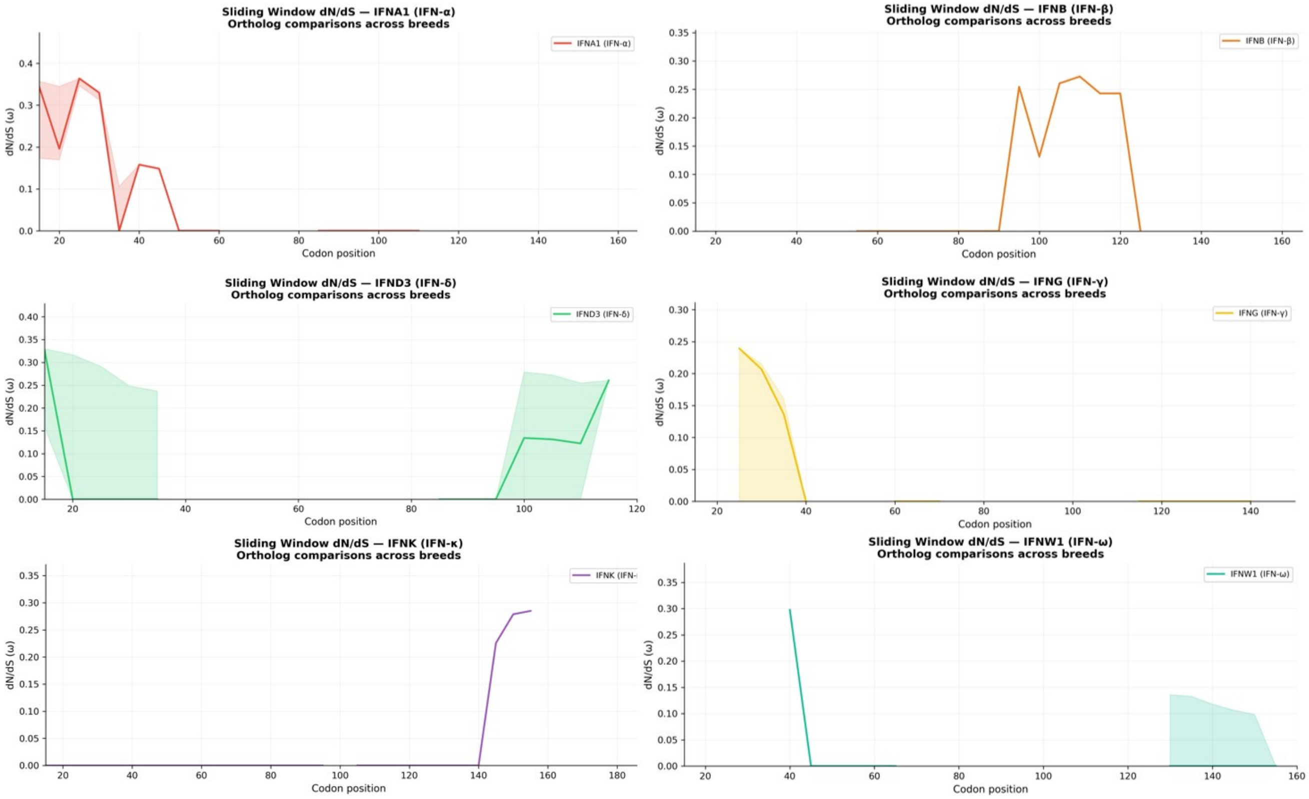

Figure S2. Sliding-window dN/dS profiles of representative porcine IFN orthologs across breeds. Sliding-window estimates of nonsynonymous to synonymous substitution ratios (dN/dS, ω) were calculated across codon positions for representative IFN orthologs, including Type I IFNs IFN-α (IFNA1), IFN-β (IFNB), IFN-δ (IFND3), IFN-κ (IFNK), and IFN-ω (IFNW1), and Type II IFN-γ (IFNG). The x-axis indicates codon position, and the y-axis indicates window-based dN/dS values. Across all six representative genes, ω values remained below 1.0, supporting predominant purifying selection across coding regions. Localized increases in ω were observed in some regions, but these peaks remained below the neutral threshold, indicating regional variation in selective constraint rather than evidence of positive selection.

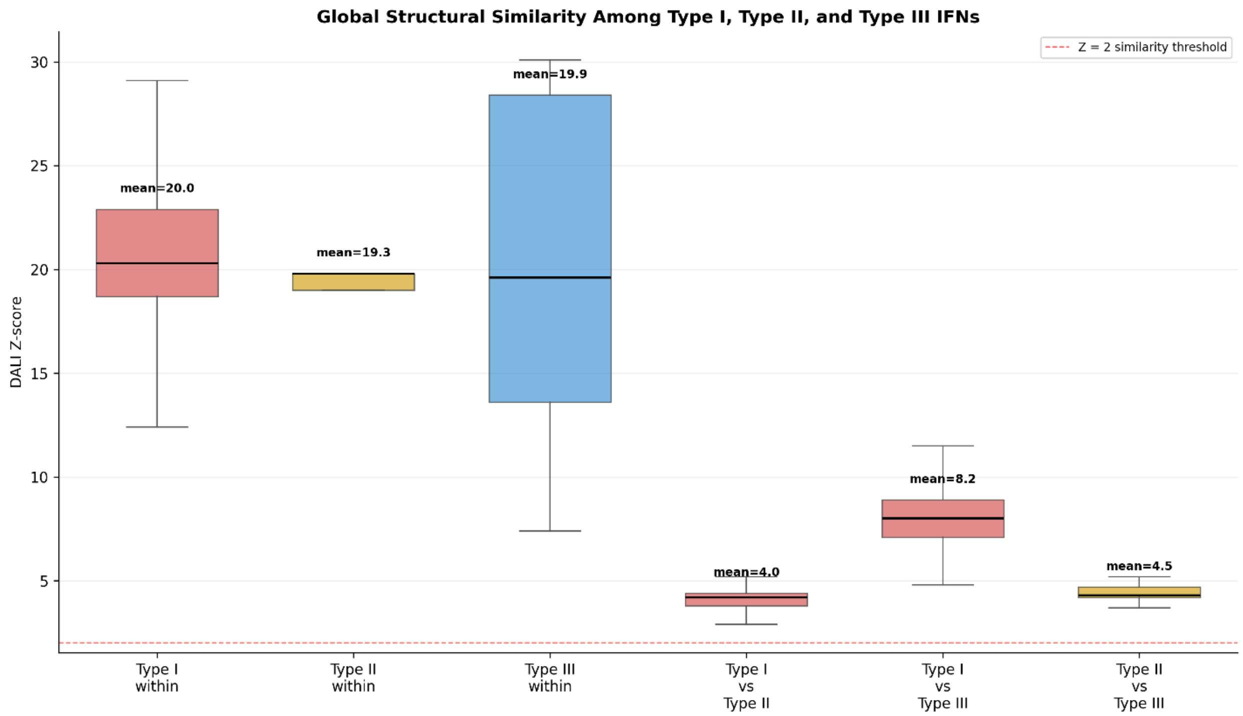

Figure S3. Global structural similarity among Type I, Type II, and Type III porcine interferons. All 432 predicted IFN structures were grouped into Type I IFNs (n = 378; IFN-α, IFN-αω, IFN-β, IFN-δ, IFN-ε, IFN-κ, and IFN-ω), Type II IFNs (n = 11; IFN-γ), and Type III IFNs (n = 43; IFN-λ). Boxplots summarize the distributions of pairwise DALI Z-scores for unique non-self comparisons within each IFN type and between the three IFN types. The central line indicates the median, boxes represent the interquartile range, and whiskers extend to values within 1.5 times the interquartile range. Mean Z-scores are shown above each box. Mean within-type Z-scores were 20.0, 19.3, and 19.9 for Type I, Type II, and Type III IFNs, respectively. Mean inter-type Z-scores were 4.0 for Type I versus Type II, 8.2 for Type I versus Type III, and 4.5 for Type II versus Type III. The red dashed line indicates the DALI structural-similarity threshold of Z = 2. The higher similarity between Type I and Type III IFNs indicates that IFN-λ retains greater global structural resemblance to Type I IFNs than either group does to IFN-γ.

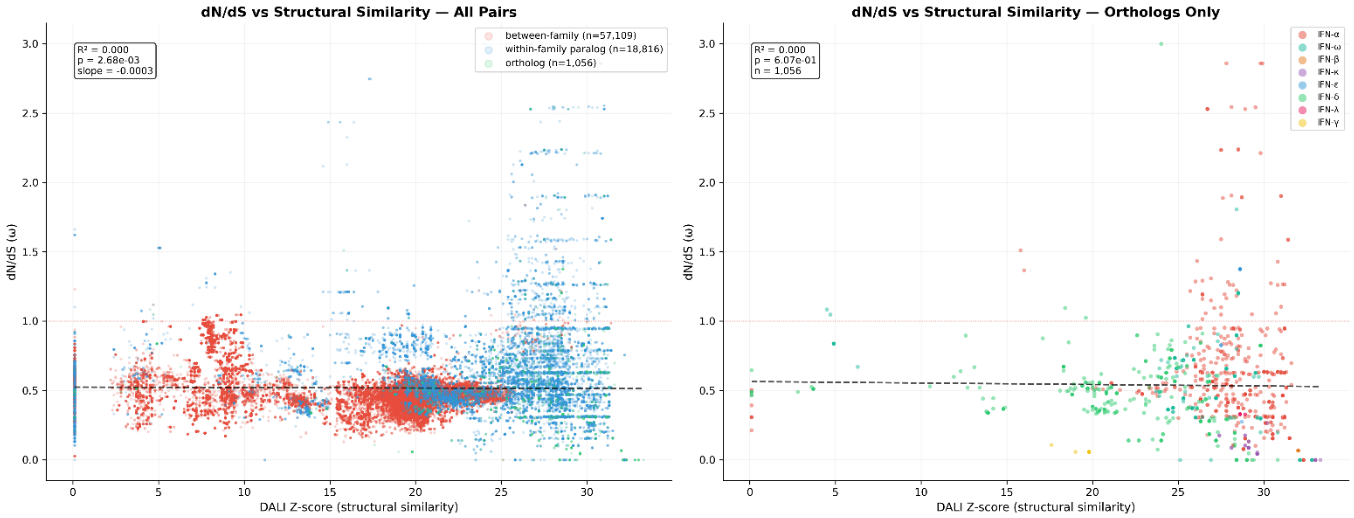

Figure S4. Relationship between sequence-level selective pressure and structural similarity across porcine IFN proteins. Pairwise dN/dS values were compared with corresponding DALI structural similarity Z-scores for 83,028 matched structure-sequence pairs. The left panel shows all matched comparisons, including between-family comparisons, within-family paralog comparisons, and cross-breed ortholog comparisons. The right panel shows only orthologous IFN comparisons across breeds, colored by IFN family. The red dashed horizontal line indicates $\omega = 1$, corresponding to neutral evolution, while values below 1 indicate purifying selection. The black dashed line indicates the fitted linear regression trend. Across all matched pairs, dN/dS showed no meaningful linear association with DALI Z-score ($R^2 \approx 0.000$; slope = −0.0003). Ortholog-only comparisons similarly showed no significant relationship between structural similarity and dN/dS ($R^2 \approx 0.000$, $p = 0.607$, $n = 1,056$). These results indicate that structural conservation and codon-level selective pressure are largely decoupled across the porcine IFN repertoire.
